# Assessing the Thiamine Diphosphate Dependent Pyruvate Dehydrogenase E1 Subunit for Carboligation Reactions with Aliphatic Ketoacids

**DOI:** 10.3390/ijms21228641

**Published:** 2020-11-16

**Authors:** Stefan R. Marsden, Duncan G. G. McMillan, Ulf Hanefeld

**Affiliations:** Biokatalyse, Afdeling Biotechnologie, Technische Universiteit Delft, Van der Maasweg 9, 2629HZ Delft, The Netherlands; S.R.Marsden@tudelft.nl (S.R.M.); D.G.G.McMillan@tudelft.nl (D.G.G.M.)

**Keywords:** Thiamine diphosphate, transketolase, C-C bond formation, kinetic control, acyloins

## Abstract

The synthetic properties of the Thiamine diphosphate (ThDP)-dependent pyruvate dehydrogenase E1 subunit from *Escherichia coli* (*Ec*PDH E1) was assessed for carboligation reactions with aliphatic ketoacids. Due to its role in metabolism, *Ec*PDH E1 was previously characterised with respect to its biochemical properties, but it was never applied for synthetic purposes. Here, we show that *Ec*PDH E1 is a promising biocatalyst for the production of chiral α-hydroxyketones. WT *Ec*PDH E1 shows a 180–250-fold higher catalytic efficiency towards 2-oxobutyrate or pyruvate, respectively, in comparison to engineered transketolase variants from *Geobacillus stearothermophilus* (TK_GST_). Its broad active site cleft allows for the efficient conversion of both (*R*)- and (*S*)-configured α-hydroxyaldehydes, next to linear and branched aliphatic aldehydes as acceptor substrates under kinetically controlled conditions. The alternate, thermodynamically controlled self-reaction of aliphatic aldehydes was shown to be limited to low levels of conversion, which we propose to be due to their large hydration constants. Additionally, the thermodynamically controlled approach was demonstrated to suffer from a loss of stereoselectivity, which makes it unfeasible for aliphatic substrates.

## 1. Introduction

**Scheme 1:**
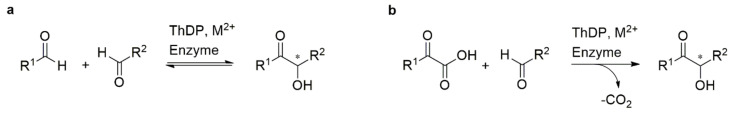
(**a**) Thermodynamically controlled conversion of aldehyde substrates. A lack of discrimination between the donor and acceptor substrate can lead to additional regioisomers, which typically limits this approach to R^1^ = R^2^. (**b**) Decarboxylation renders the reaction kinetically controlled and allows for mixed carboligation reactions (R^1^ ≠ R^2^) in a controlled fashion. Monitoring of the reaction’s progress is required to prevent subsequent thermodynamic equilibration towards the corresponding aldehyde starting materials [5,6].

Thiamine diphosphate (ThDP) dependent enzymes are excellent biocatalysts for the synthesis of chiral α-hydroxyketones (acyloins) from two aldehydes [1,2]. The reaction is initiated by the activated ThDP cofactor forming a covalent intermediate with the donor substrate. This induces an ‘Umpolung’ that turns the carbonyl group into a nucleophile [3,4]. In spite of being 100% atom efficient, unfavourable equilibrium conditions can limit this thermodynamically controlled approach to low levels of conversion [5]. This issue can be addressed via the decarboxylation of ketoacids as donor substrate analogues, which renders the reaction kinetically controlled and allows for complete conversion (Scheme 1) [5,6]. This feature makes the application of ThDP-dependent enzymes interesting for industrial applications [7]. Their acceptor substrate scope was engineered to allow for the conversion of aliphatic- [5,8,9], aromatic- [10] and non-phosphorylated [11,12] aldehyde substrates with enhanced or reversed stereoselectivity [13,14,15], of which comprehensive reviews were published elsewhere [16,17,18]. Yet, the expansion of the donor substrate scope by mutagenesis remains a formidable challenge.

A multitude of essential amino acid interactions are required for the binding and activation of the ThDP cofactor and must not be disrupted if function is to be retained [19]. A metal binding site coordinates a divalent cation (typically Mg^2+^), which ionically binds the ThDP cofactor via its pyrophosphate group. A distal glutamate and histidine residue then function together as a catalytic base to convert the cofactor into its active ylide state via deprotonation of the thiazole ring [20,21]. To spatially allow for the required proton transfer, the ThDP cofactor adopts an energetically disfavoured V-conformation, a feature that is evolutionary conserved within the class of ThDP-dependent enzymes [22,23,24]. Additional interactions with the covalent intermediate further promote the distorted conformation and prevent its relaxation into a lower energy state. The activation energy for the following C-C bond formation is thereby reduced [25]. The holoenzyme is usually a homodimer with two symmetrical active sites located at the dimer interface. The rate of holoenzyme formation, ThDP affinity, and extent of cooperativity strongly depend on the interplay between the divalent metal and the ThDP cofactor [26,27,28,29,30,31,32,33,34,35]. A proton wire allows for communication between the two active sites, making them non-equivalent in terms of cofactor affinity and affects an alternating half-of-the-sites reactivity [32,33,36,37]. A switch from positive, cooperative binding of ThDP (*n* ≈ 2 at 1 mM Mg^2+^) to negative cooperativity (*n =* 0.61 at 3 mM of Mg^2+^) highlights the complexity of interactions in *E. coli* TK [34].

Furthermore, oxidative stress during protein expression was shown to result in the post-translational oxidation of Cys157 to a sulfenic acid group in *E. coli* TK. This oxidation leads to a 100-fold increased affinity towards ThDP and a 20-fold increase in transketolase activity [34].

**Figure 1:**
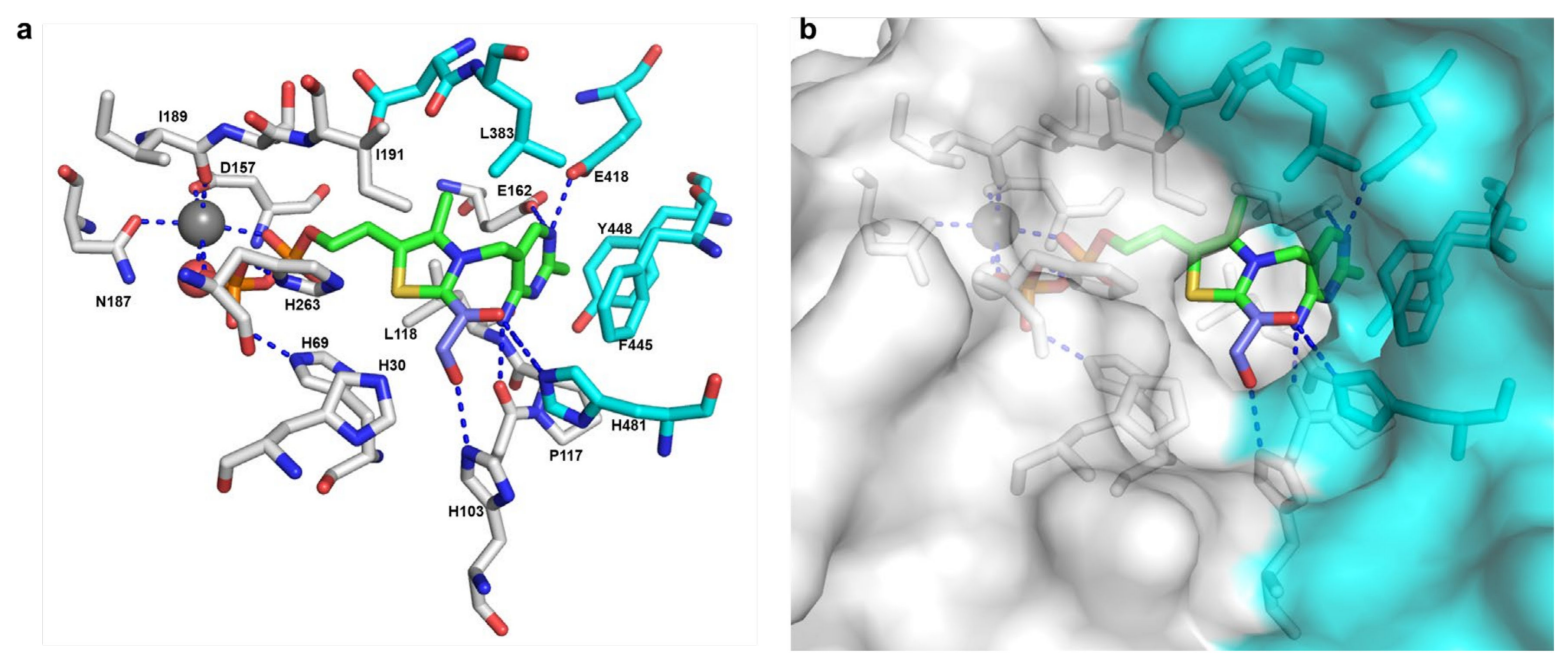
(**a**) Interactions between the Thiamine diphosphate (ThDP) cofactor (green) and residues from both monomers (white/cyan) in *Saccharomyces cerevisiae* transketolase (*Sc*TK). The covalent C_2_-ketol intermediate is stabilised by hydrogen bonds with two histidine residues. (**b**) Surface view of the active site in WT *Sc*TK. The ThDP cofactor is buried within the enzyme, and only accessible via a narrow substrate channel. This sterically limits the size of the donor substrate to the transfer of a C_2_-ketol unit in WT *Sc*TK. Figures were created in PyMol from 1gpu.pdb. Dotted lines denote H-bonds or ionic interactions at 2–4 Å distance.

Examples for the conversion of non-natural donor substrates by ThDP-dependent enzymes are scarce, due to the highly specific interactions within their active sites (Figure 1). This feature constitutes a major drawback in terms of engineering, as it limits the structural diversity that is currently accessible with ThDP dependent enzymes. Examples include the decarboxylative conversion of (*S*)-4-hydroxy-2-oxoglutarate with the enzyme 2-succinyl-5-enolpyruvyl-6-hydroxy-3-cyclohexene-1-carboxylate synthase (MenD) from *E. coli* using a kinetically controlled approach [38], while thermodynamically controlled one-substrate, benzoin-type reactions were demonstrated with benzaldehyde lyase (BAL) from *Pseudomonas fluorescens biovar I* using benzaldehyde derivatives [39]. Notably, a thermostable transketolase from *Geobacillus stearothermophilus* (TK_GST_) was successfully engineered by employing successive rounds of iterative site-saturation mutagenesis towards the conversion of pyruvate, 2-oxobutyrate and 3-methyl-2-oxobutyrate as donor substrates [40], of which triple variants were recently reported in a follow-up study [41]. Similarly, *E. coli* TK was also engineered for the conversion of pyruvate [42].

However, transketolases naturally evolved to specifically accept highly polar, phosphorylated carbohydrates as natural substrates [43], which makes their wild-type variants inept for the conversion of aliphatic ketoacids (e.g., pyruvate). For this reason, engineered TK variants still tend to display rather low catalytic efficiencies towards these non-natural substrates [40,41,42]. Since ThDP dependent enzymes comprise a modular structure and share the same catalytic mechanism [44], we hypothesised that the screening of different enzyme scaffolds could prove more suitable for the conversion of aliphatic ketoacids in carboligation reactions.

**Figure 2:**
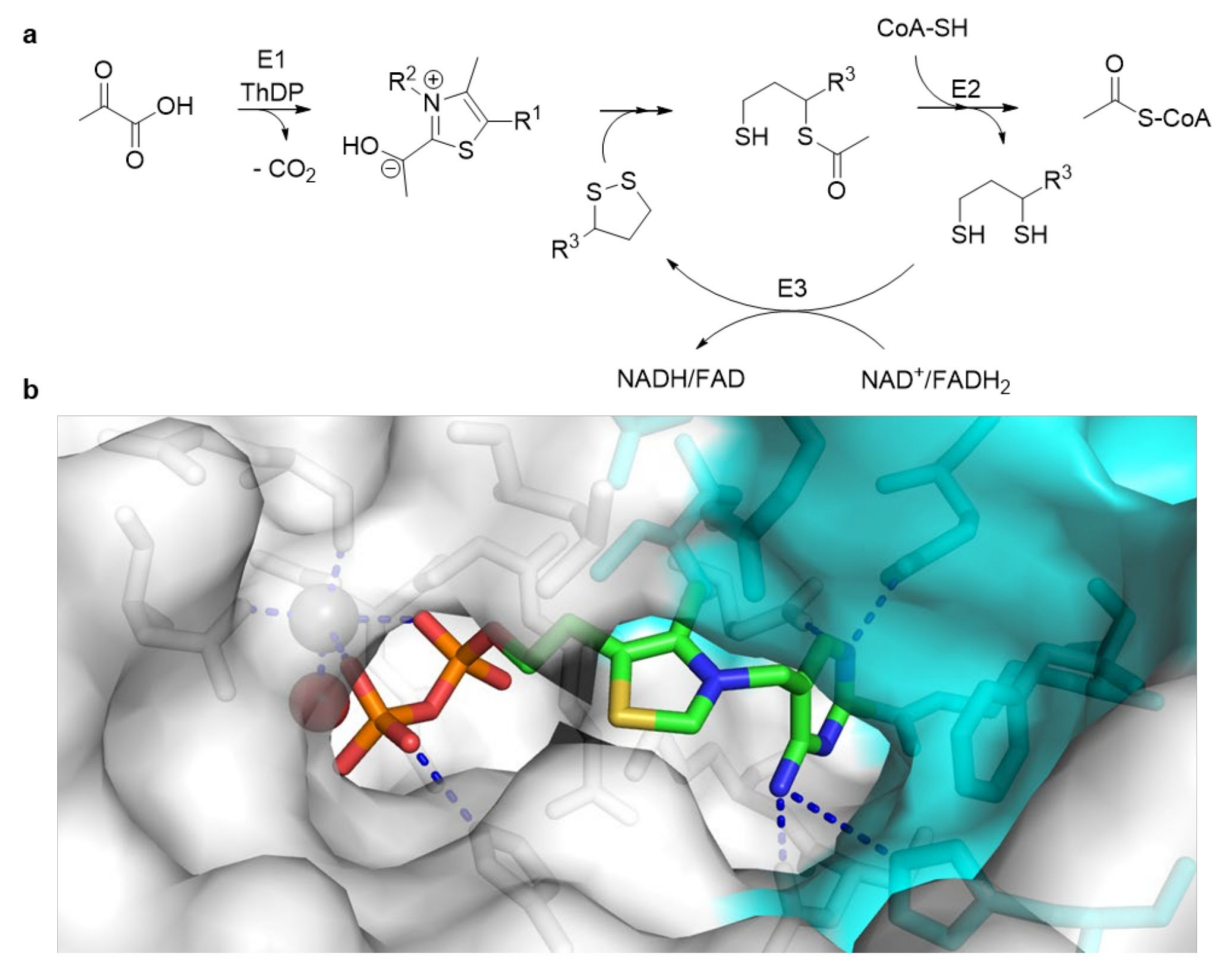
(**a**) Natural reaction of the pyruvate dehydrogenase complex (PDHc): ThDP-dependent E1 forms a covalent intermediate with pyruvate and catalyses its decarboxylation. Subsequent C-S bond formation with a bulky lipoate moiety affords a thioester. Transesterification by E2 then affords acetyl-CoA, and Flavin-mediated oxidation by E3 closes the catalytic cycle. (**b**) Surface view of the active site in WT *Ec*PDH E1. A broad active site cleft allows for the accommodation of sterically more demanding substrates. Figures were created with PyMol from 2iea.pdb.

The Thiamine Enzyme Engineering Database (TEED) provides an excellent overview of the nine superfamilies of ThDP-dependent enzymes, including sequence and structural data [45,46,47]. During a qualitative database search, the ThDP-dependent pyruvate dehydrogenase E1 subunit from *E. coli* (*Ec*PDH E1) attracted our attention. As part of the pyruvate dehydrogenase complex (PDHc), the E1 subunit catalyses the decarboxylative activation of pyruvate for the synthesis of acetyl-CoA (Figure 2a) [48]. Due to its relevance for metabolism, E1 was extensively characterised with respect to its biochemical properties, but has never been applied for synthetic purposes [49].

*Ec*PDH E1 does not require the presence of the E2 and E3 subunits and purified *Ec*PDH E1 is fully active alone [50]. With pyruvate as its natural donor substrate, *Ec*PDH E1 evolved towards the efficient conversion of aliphatic ketoacids and shows an inherent promiscuity towards 2-oxobutyrate [51]. The use of a sterically demanding lipoate moiety as its natural acceptor substrate requires a wide substrate channel, and should therefore enable the conversion of a broad range of substrates (Figure 2b). This is in stark contrast to the narrow substrate channel of transketolases (Figure 1b). Notably, this also implies that *Ec*PDH E1 should not display a requirement for phosphorylated substrates; a feature which commonly impairs the catalytic efficiency of transketolases [11]. These properties motivated us to assess the synthetic performance of *Ec*PDH E1 for the conversion of aliphatic ketoacids in carboligation reactions.

## 2. Results and Discussion

### 2.1. Characterisation of WT EcPDH E1

#### 2.1.1. Expression and Purification

The *aceE* gene (encoding for *Ec*PDH E1, accession number P0AFG9, EC 1.2.4.1) was codon optimised for recombinant expression in *E. coli*. The target gene was subsequently cloned into the pBAD/HisA expression plasmid using the designed KpnI and XhoI restriction sites. This cloning strategy introduced an *N*-terminal His_6_-tag, which is separated from the *N*-terminus by a linker of 32 amino acids.

*E. coli* Top10 cells were transformed with the final construct, and the enzyme was expressed in a batch fermentation until an increase in dissolved oxygen indicated the depletion of nutrients (Figure S1). *Ec*PDH E1 was subsequently purified by affinity chromatography via its *N*-terminal His_6_-tag to give a pure protein yield of 350 mg/L of expression medium under suboptimal conditions (Figures S2 and S3). Notably, the disruption of cells by ultrasonication on ice led to a complete loss of activity, while an active enzyme was obtained with a cell disrupter using three passes at 1.8 kbar in combination with lysozyme.

#### 2.1.2. Optimisation of Reaction Conditions

**Figure 3:**
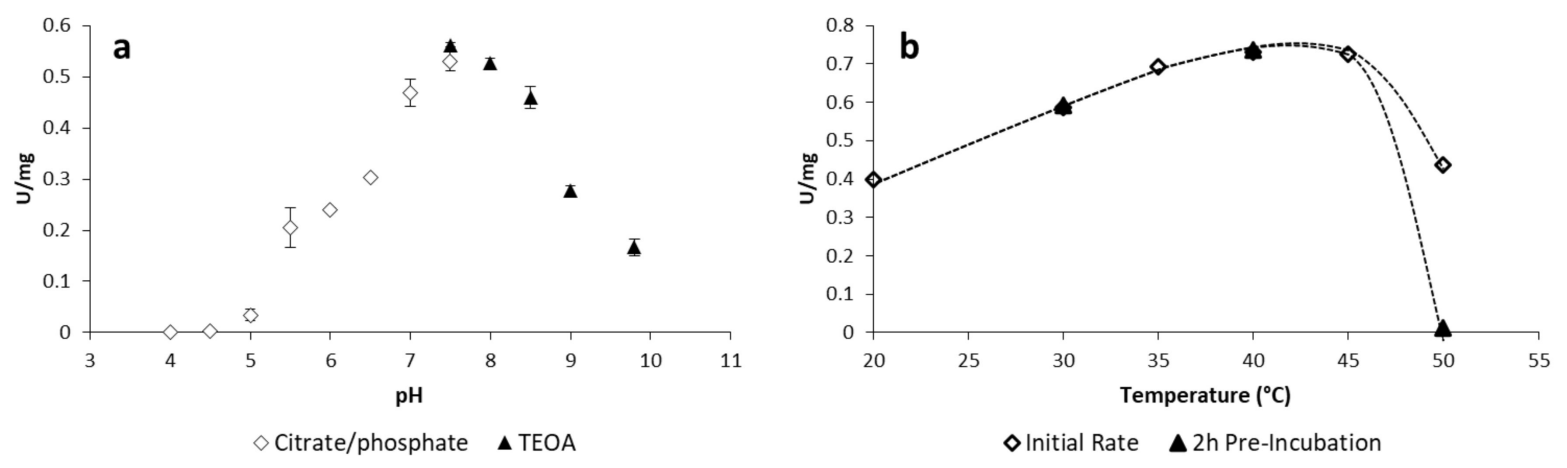
(**a**) A pH optimum of 7.5 was determined for *Ec*PDH E1 activity by the DCPIP assay (0.2 mM ThDP, 2 mM MgCl_2_, 2mM sodium pyruvate, 100 µM DCPIP, 20 mM KPi, 25 °C). (**b**) *Ec*PDH E1 displays a maximum activity at 40 °C with no concomitant loss of activity after two hours. Higher temperatures swiftly reduce the initial rate and lead to a complete inactivation (0.2 mM ThDP, 2 mM MgCl_2_, 2 mM sodium pyruvate, 100 µM DCPIP, 20 mM KPi, pH 7.5). Control reactions were performed in the absence of an enzyme.

A spectrophotometric assay utilising 2,6-dichloroindophenol (DCPIP) and pyruvate as substrates (Scheme S1) was initially used to identify optimal conditions for *Ec*PDH E1 catalysed reactions [52]. The reduction in DCPIP was followed at its isosbestic point (517 nm, Figures S4 and S34) to allow for the determination of the optimal pH. E1 retained its activity over a broad pH range from pH 5.5 to 9.5, with its highest activity at pH 7.5 (Figure 3a). The enzyme showed good thermal stability with no loss of activity after two hours of incubation at 40 °C. However, higher temperatures swiftly lead to its complete inactivation (Figure 3b). Notably, *Ec*PDH E1 can be stored with no loss of activity for at least six months at −20 °C (5 mg/mL *Ec*PDH E1 in 20 mM potassium phosphate buffer, pH 7.0).

#### 2.1.3. Preparative Scale Reactions

**Scheme 2:**
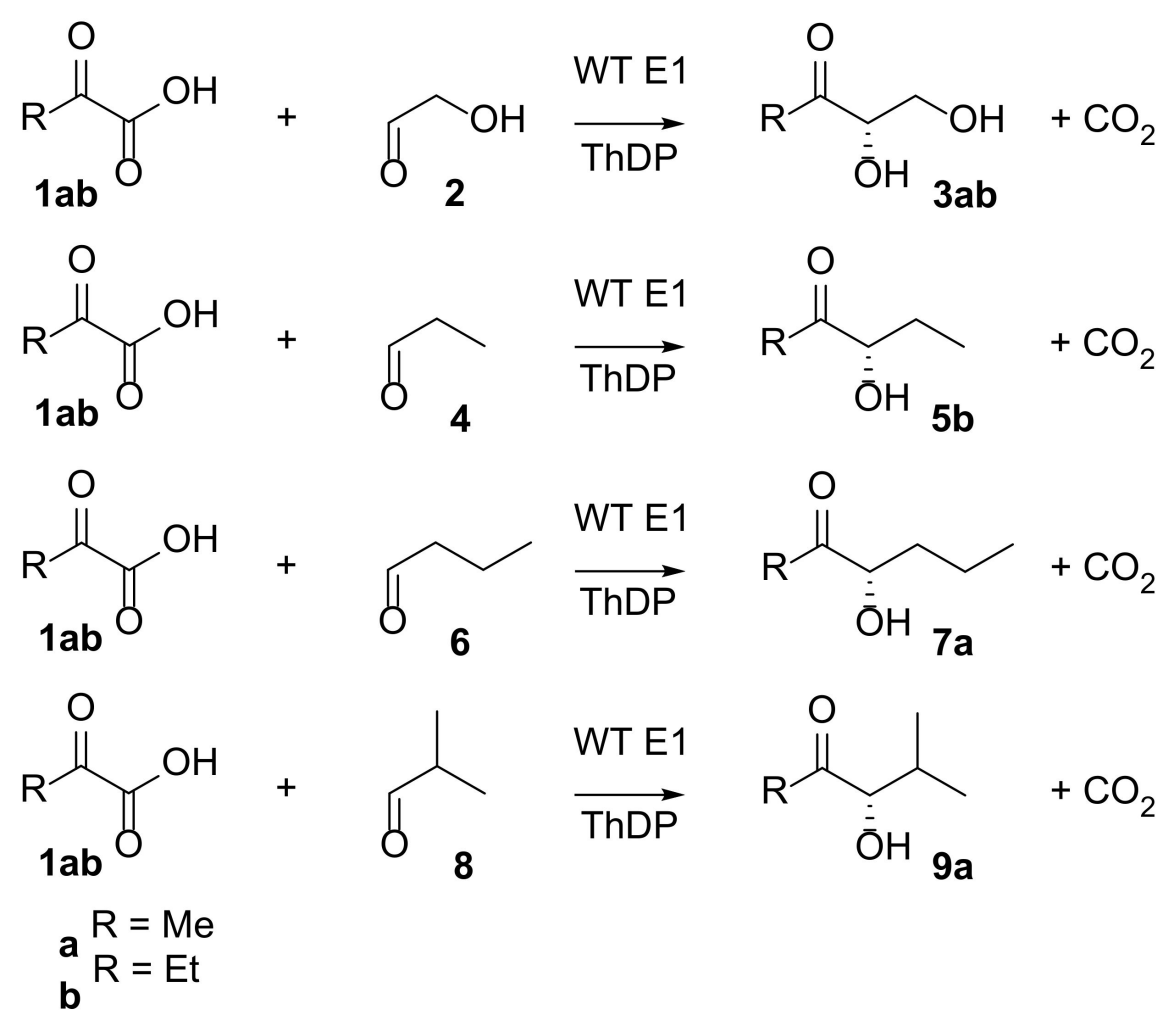
Preparative scale (0.6–10 mmol) coupling of ketoacids **1ab** with various aldehydes to afford the corresponding (*S*)-configured acyloins under kinetically controlled conditions.

Having identified optimal conditions for *Ec*PDH E1 catalysed reactions, we set out to investigate its substrate scope on a preparative scale. A small excess of 1.2 equivalents of either the donor or the acceptor substrate was used where appropriate, in order to benefit the subsequent workup by extraction. Both pyruvate and 2-oxobutyrate were readily accepted as donor substrates. Regarding the acceptor scope, polar α-hydroxyaldehydes were efficiently converted next to apolar, linear and branched aliphatic aldehydes (Scheme 2). *Ec*PDH E1 displayed (*S*)-selectivity, and the corresponding acyloins were consistently obtained in good enantiomeric purity (93–95% *ee*) and poor to good yields (5–70%, Table 1) due to their volatility. Similar enantiomeric purities were reported for engineered TK_GST_ variants using α-hydroxyaldehyde acceptor substrates [40]. However, the combination of 2-oxobutyrate as a donor substrate with aliphatic aldehyde acceptors previously led to an almost complete loss of stereoselectivity (6–33% *ee*) in TK_GST_ variants [41], which can now be produced in good enantiomeric purity by *Ec*PDH E1. The isolated products were subsequently used as external standards for a more detailed kinetic analysis of WT *Ec*PDH E1 by HPLC (Figures S35–S42).

#### 2.1.4. Conversion of (*R*)- and (*S*)-Configured α-hydroxyaldehyde Acceptor Substrates

Transketolases catalyse the kinetic resolution of α-hydroxyaldehydes, displaying a strong stereopreference for the (*2R*)-configuration [53,54]. While this may be desirable in some cases, this feature prevents the conversion of (*2S*)-configured substrates and limits the product scope when enantiopure substrates are available.

To investigate a possible stereopreference regarding the configuration of α-hydroxyaldehyde substrates, the reaction between racemic DL-glyceraldehyde and pyruvate was followed over time (Figure S5). Complete conversion of the racemic substrate was readily achieved, and the time course did not indicate a notable discrimination between D- and L-glyceraldehyde. With lipoate as its natural acceptor substrate, *Ec*PDH E1 possesses a broad active site cleft that does not require residues for the recognition of hydroxyl groups. E1 therefore displays an extended product scope over transketolases by allowing for the efficient conversion of both (*R*)- and (*S*)-configured α-hydroxyaldehydes.

#### 2.1.5. Thermodynamically Controlled One-Substrate Reactions

**Figure 4:**
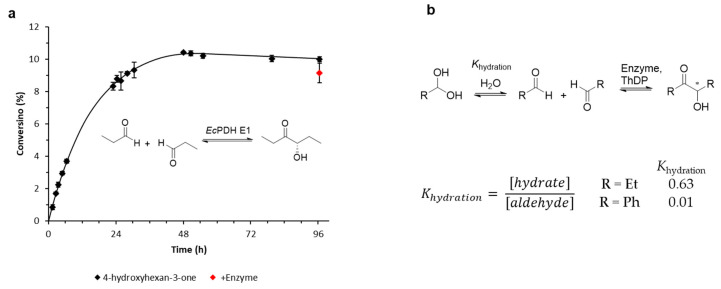
(**a**) Time course of the *Ec*PDH E1 catalysed self-reaction of propionaldehyde (2 mg/mL *Ec*PDH E1, 0.2 mM ThDP, 2 mM MgCl_2_, 50 mM propionaldehyde, 20 mM KPi, pH 7.5). The position of the thermodynamic equilibrium limits the reaction to 10% conversion under given experimental conditions. (**b**) The hydration of aldehydes in aqueous solution introduces a competing equilibrium reaction. Electrophilic aldehydes display a large hydration constant [56], which renders the target reaction thermodynamically less favourable.

Benzaldehyde lyase (BAL) catalyses the thermodynamically controlled coupling of aromatic aldehydes in benzoin-type reactions, which proceeds with 100% atom economy and was reported to afford the products in both high enantiomeric purity and yield [39]. Similarly, transketolase catalyses the self-reaction of glycolaldehyde to erythrulose [55], albeit with low conversion due to an unfavourable equilibrium constant [5]. With this in mind, the efficiency of *Ec*PDH E1 to catalyse the self-reaction of aliphatic aldehydes was explored. (*4S*)-hydroxyhexan-3-one **5b** can be synthesised from 2-oxobutyrate and propionaldehyde under kinetically controlled conditions, so its alternate synthesis via the self-reaction of propionaldehyde was also examined. The reaction was found to converge towards 10% conversion, and equilibrium conditions were demonstrated by the addition of extra enzyme; no change was observed (Figure 4). The examples of *Sc*TK and *Ec*PDH E1 catalysed self-reactions are in stark contrast to the performance of BAL catalysed conversions with aromatic aldehydes, where high yields were reported [39]. Notably, aldehydes can form hydrates in aqueous solution, and their hydration constant is determined by the electrophilicity of the carbonyl group. This hydration of aldehydes constitutes a competing side reaction, which influences the maximal extent of conversion. Aromatic aldehydes are stabilised through resonance, and the hydration of benzaldehyde is only minor. In contrast, propionaldehyde shows a 63-fold higher hydration constant, which further increases with the presence of electron withdrawing residues (e.g., a hydroxyl group in glycolaldehyde) [56]. The viability of thermodynamically controlled self-reactions of aldehydes with respect to maximal conversions can therefore readily be assessed from published hydration constants.

**Figure 5:**
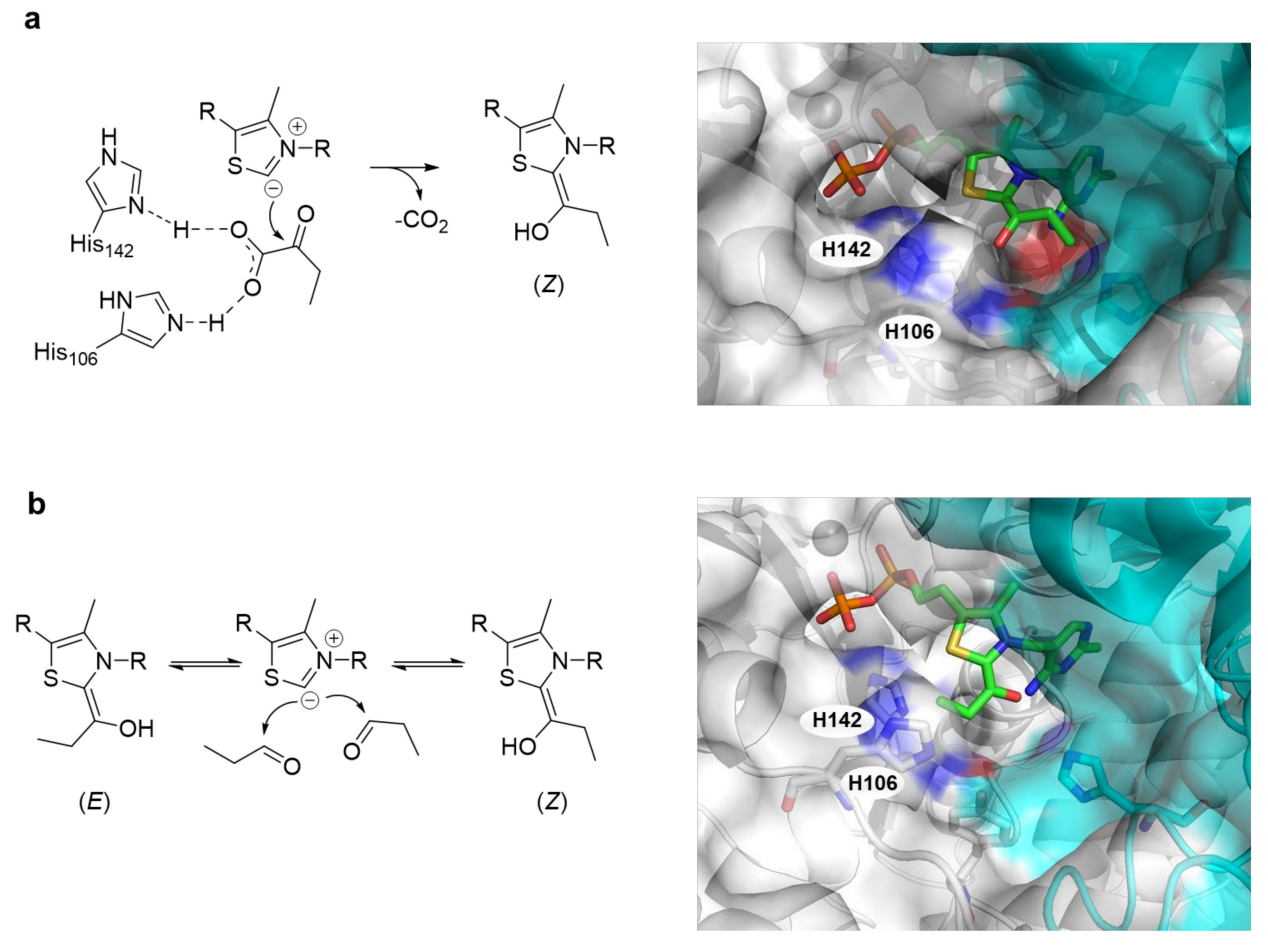
(**a**) The presence of two histidine residues (H106 and H142, surface polarity shown in blue) presumably guides the approach of negatively charged ketoacid substrates into the active side. This prearrangement could lead to the preferential formation of the (*Z*)-configured intermediate upon decarboxylation. (**b**) Modelling suggests that the smaller substrate propionaldehyde can approach the cofactor on different trajectories, which may lead to the formation of both (*E*) and (*Z*)-configured intermediates. This could influence the orientation of the acceptor substrate and give rise to both (*R*) and (*S*)-configured acyloin products. Models and figures were created with PyMol from 2iea.pdb.

Curiously, an enantiomeric excess of only 67% was obtained after 24 h for the synthesis of **5b** under thermodynamic control. Conversely, decarboxylation of 2-oxobutyrate as substrate analogue afforded **5b** with 95% *ee* under kinetic control, using the same catalyst loading and reaction time. Enzyme catalysed racemization therefore seems an unlikely explanation for this observation. Since propionaldehyde functions as acceptor substrate in both cases, the disparity in stereocontrol was assumed to arise from the two different mechanisms of formation for the covalent intermediate [5,6]. We therefore analysed the crystal structure of holo-*Ec*PDH E1 (2iea.pdb) and created models for possible configurations of the covalent intermediate in silico (Figure 5). The covalent bond between the ThDP cofactor and the donor substrate has double bond character through resonance, which impairs its rotation and favours a more planar configuration (see Figure 1 for the ketol intermediate in TK). The requirement to accommodate a bulky lipoate moiety as an acceptor prevents the recognition of small donor substrates via sterical constraints, and *Ec*PDH E1 presumably evolved to guide the approach of pyruvate towards the cofactor via ionic interactions. Two histidine residues (H106 and H142) are located on the left side and allow for interactions with negatively charged ketoacids. Such a prearrangement would preferentially give rise to the (*Z*)-conformation in the covalent intermediate (Figure 5a). Our model suggests that the smaller and uncharged propionaldehyde could approach the cofactor on different trajectories, which would give rise to both (*Z*)- and (*E*)-conformers (Figure 5b). The substantial geometrical difference between the (*E*)- and (*Z*)-configurations would plausibly result in different orientations of the acceptor substrate towards the carbanion, leading to the formation of both (*R*)- and (*S*)-configured products. The synthetic performance of thermodynamically controlled reactions catalysed by ThDP dependent enzymes may therefore not only differ with respect to maximum yields, but also the obtained enantiomeric purities.

#### 2.1.6. Determination of Kinetic Parameters

In order to compare the catalytic performance of WT *Ec*PDH E1 with other (engineered) ThDP-dependent enzymes, kinetic parameters were determined for different donor and acceptor substrates by HPLC (Table 2, Figures S18–S23). Wild-type *Ec*PDH E1 showed a 250-fold higher catalytic efficiency for the conversion of pyruvate than the best engineered variant of TK_GST_ after two rounds of iterative site-saturation mutagenesis [40]. Notably, this was also 16-fold higher than the catalytic efficiency of WT *Ec*DXS, which similarly uses pyruvate as its natural donor substrate [40]. For 2-oxobutyrate, WT *Ec*PDH E1 showed a 180-fold higher catalytic efficiency than the TK_GST_ double variant H102L/H474S. Conversely, E1 proved to be a poor catalyst for the conversion of hydroxypyruvate, where it was outperformed by (engineered) variants of transketolase by three orders of magnitude.

Regarding the acceptor substrates, transketolases exhibit features for the recognition of phosphorylation and the configuration of hydroxyl groups in their natural substrates [43,53,54], which previously needed to be removed by mutagenesis for the efficient conversion of non-natural substrates [5,11,12,57]. Here, WT *Ec*PDH E1 displayed a specific activity towards both α-hydroxyaldehydes and aliphatic aldehydes that was comparable to the engineered transketolase variants.

Notably, different variants were evolved at varying positions for the conversion of either aliphatic donor or acceptor substrates, respectively, which may not be mutually compatible. In summary, transketolases naturally evolved towards highly polar, phosphorylated carbohydrates. This makes their wild-type scaffolds inept for the conversion of aliphatic ketoacids. While moderate improvements can be achieved by rational mutagenesis, these variants are still considerably outperformed by wild-type enzymes such as *Ec*DXS [40] and *Ec*PDH E1 (this study).

### 2.2. Mutagenesis

**Figure 6:**
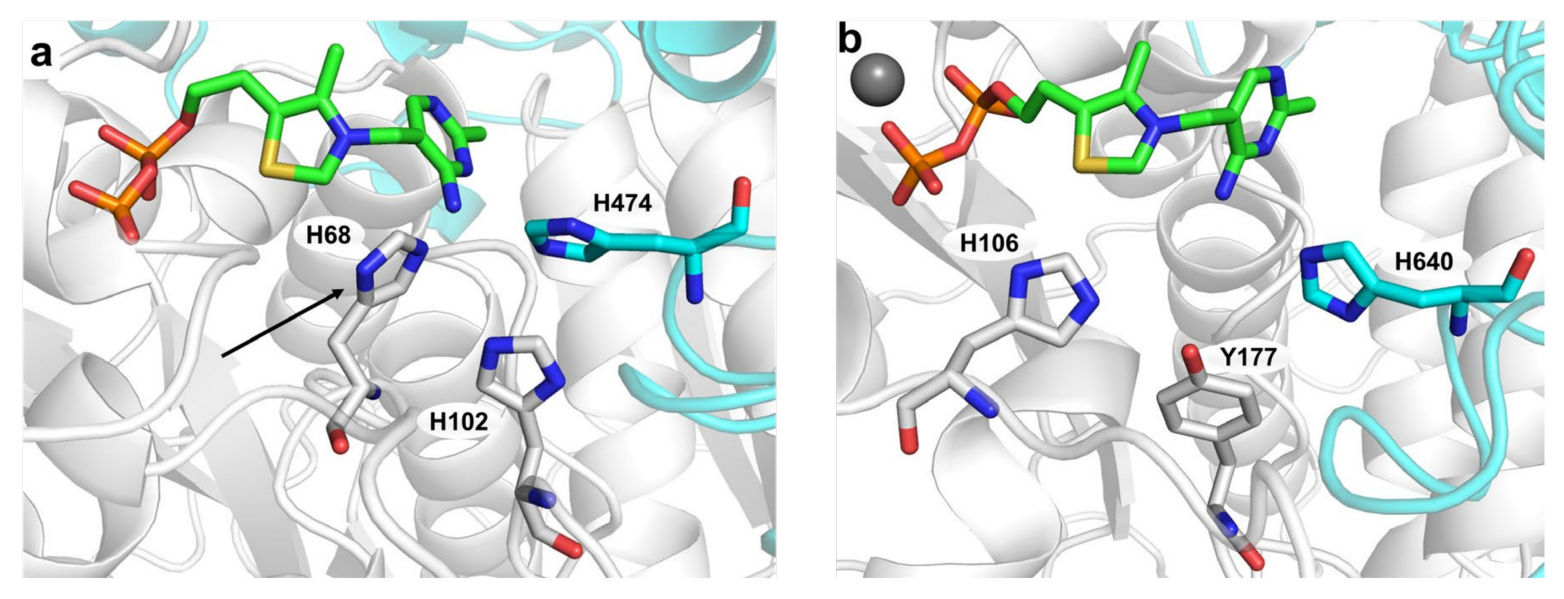
(**a**) Active site view of the homology model for TK_GST_. Residue H68 is essential for the activation of ThDP, whereas residues H102 and H474 were successfully mutated towards the conversion of aliphatic ketoacids [40]. (**b**) Active site view of *Ec*PDH E1 (2iea.pdb). The position of H102 is occupied by Y177 as the most prominent difference between the active sites of TK_GST_ and *Ec*PDH E1. Residues Y177 and H640 were therefore similarly targeted for mutagenesis, based on the most successful variants that were previously identified in TK_GST_. The homology model was created with YASARA, figures were created with PyMol.

While TK_GST_ variants also showed some promiscuity towards the sterically more challenging donor substrate 3-methyl-2-oxobutyrate (0.004–0.012 s^−1^mM^−1^) [40], no conversion was observed with WT *Ec*PDH E1. Due to the absence of a crystal structure for TK_GST_ a homology model was created in YASARA. This model was then compared to the crystal structure of WT *Ec*PDH E1 (2iea.pdb, Figure 6). Both active sites are largely comprised of the same conserved residues, but their overall structural dimensions differ considerably (Figure S17). Most notably, distal tertiary and quaternary structural features place the loop bearing residue H68 in TK (corresponding to H106 in E1) deeper into the active site of TK. This is not the case in *Ec*PDH E1, which allows its active site to be overall more spacious (Figure 1, Figure 6 and Figure S17). Notably, residue H102 in TK_GST_ is replaced by Y177 in *Ec*PDH E1 as the most prominent difference in active site residues. The bulkiness of Y177 could also provide a plausible explanation for why the branched substrate 3-methyl-2-oxobutyrate was not converted by WT E1. Residue H68 is essentially conserved in TK_GST_ due to its role in cofactor binding and activation; however, mutations at H474 (S,N) and H102 (G,L,T) enabled the conversion of aliphatic ketoacids by TK_GST_ [40]. Most of these variants are capable of forming hydrogen bonds via their amino-acid side chains in order to retain essential hydrogen bond interactions within the active site. Following this train of thought, we therefore chose to introduce serine residues as the smallest hydrogen bond donors at the corresponding positions (Y177S, H640S and their combination to Y177S/H640S) in *Ec*PDH E1. While all three variants retained their activity towards pyruvate, H640S no longer showed activity towards 2-oxobutyrate, and none of the three variants displayed any observable promiscuity towards either branched 3-methyl-2-oxobutyrate or linear 2-oxovalerate. While *Ec*PDH E1 constitutes a good scaffold for the conversion of 2-oxobutyrate, extensive site-saturation mutagenesis is required to further broaden its substrate scope. The expansion of the donor substrate scope of ThDP-dependent enzymes therefore remains a challenging field.

## 3. Materials and Methods

### 3.1. pH Depend. Activity

(*n* = 3) In [52]: 2,6-dichloroindophenol (DCPIP) was dried under reduced pressure in a desiccator over night before preparation of a stock solution (10 mM, in 20 mM KH_2_PO_4_, pH = 7.0). The isosbestic point (517 nm) was determined by measuring absorbance spectra at three different pH values (3.8, 6.3 and 8.3) and a calibration curve was prepared over a range of 0-100 µM (517 nm: ε = 3.74 mM^−1^cm^−1^, R^2^ = 0.999). Reaction mixtures containing 0.2 mM ThDP, 2 mM MgCl_2_, 2 mM sodium pyruvate and 100 µM DCPIP were incubated at 25 °C. The reaction was initiated by the addition of 100 µg of holo-*Ec*PDH E1 and the decrease in absorbance was followed at the isosbestic point (517 nm, 500 rpm) in triplicate in polystyrene cuvettes on a Cary60 UV-Vis spectrometer (Agilent Technologies, Amstelveen, The Netherlands) equipped with a TC1 stirring unit (Quantum Northwest, Liberty Lake, WA, USA).

### 3.2. Temperature Dependent Activity

(*n* = 3) In [52]: temperature dependent specific activities were determined using an adapted version of the above DCPIP assay. The reaction mixture was incubated at the target temperature for 5 min before the addition of holoenzyme. The change in absorbance was recorded at the absorbance maximum (605 nm, ε = 9.01 mM^−1^cm^−1^, R^2^ = 0.999) for solutions of DPCIP at pH 7.5 for an enhanced sensitivity. Aliquots of the holoenzyme were incubated at the respective temperatures (30 °C, 120 min; 40 °C, 145 min; 50 °C, 150 min, 500 rpm) and their respective initial rates were determined in the same manner.

### 3.3. Preparative Scale Reactions

Detailed protocols are described in the supplementary information.

### 3.4. General RP-HPLC Method

Samples were quenched by 1:1 dilution with 0.2% (*v/v*) aqueous trifluoroacetic acid, and precipitated protein was removed by centrifugation (13,000 rfc, 2 min). Samples were analysed by RP-HPLC using an ICSep ICE Coregel-87H3 column (0.4 × 25 cm, Transgenomic) with 0.1% *v/v* trifluoroacetic acid as mobile phase, 0.8 mL/min, 60 °C, detection at 210 nm) on a Shimadzu LC-20AD system (Shimadzu, Kyoto, Japan). Concentrations were determined using external standards.

### 3.5. Chiral GC Methods

Chiral separation of α-hydroxyketone enantiomers was achieved by chiral phase GC using previously developed methods (Table S2): [58] 1 µL of sample (split 1/150) was analysed using a chiral CP-Chirasil-DEX CB column (Agilent, 25 m × 0.25 mM × 0.25 µm) on a Shimadzu 2010 Plus GC instrument equipped with an AOC-20i autosampler. Helium was used as carrier gas with a linear flow of 30 cm/sec. Injector temperature: 250 °C, FID-detector temperature: 275 °C.

### 3.6. Michaelis-Menten Analysis

(*n* = 2) Reaction mixtures were prepared in potassium phosphate buffer (20 mM, pH = 7.5) containing 0.2 mM ThDP, 2 mM MgCl_2_ and 0.1 mg/mL of *Ec*PDH E1. The substrate and enzyme solutions were brought to the target temperature (37 °C, 5 min) before they were mixed to initialise the reaction in final volume of 1 mL. The concentration of the second substrate was kept constant at a concentration that allowed for maximum rates. Reaction times were chosen to remain below 20 % conversion for credible initial rate conditions. Samples were analysed by RP-HPLC. Curve fitting was performed with the programme Igor using the Michaelis-Menten equation.

### 3.7. Time Course for the Conversion of Racemic DL-Glyceraldehyde with Pyruvate

*Ec*PDH E1 (0.2 mg) was incubated with ThDP (0.2 mM) and MgCl_2_ (2 mM) in potassium phosphate buffer (2 mL final volume, 20 mM KPi, pH 7.5) for 5 min, after which sodium pyruvate (50 mM) and DL-glyceraldehyde (50 mM) were added. Progress of the reaction was monitored by HPLC.

### 3.8. Homology Modelling

A homology model was built for TK_GST_ with YASARA version 19.9.17 [59] using its protein sequence [40] and the following parameters as input: PSI-BLAST iterations: 3; templates: 5; E-value 0.1.

## 4. Conclusions

In summary, our results show that WT *Ec*PDH E1 is a promising biocatalyst for the chiral synthesis of diverse acyloins under kinetically controlled conditions. The enzyme can be prepared in a high volumetric yield of 350 mg pure protein per litre of expression medium under non-optimised conditions, and shows excellent storage stability. *Ec*PDH E1 displayed a 180- to 250-fold higher catalytic efficiency than engineered transketolase variants for the conversion of pyruvate and 2-oxobutyrate, respectively. Additionally, *Ec*PDH E1 consistently showed a good stereoselectivity towards both aliphatic and hydroxyaldehyde substrates. The use of lipoate as its natural acceptor substrate requires an unusually broad active site cleft, that permits the efficient conversion of both linear and branched aliphatic aldehydes, next to polar α-hydroxyaldehydes. Notably, the absence of a stereopreference with respect to the configuration of α-hydroxyaldehydes facilitates the conversion of both enantiomers, leading to a broader product scope. Taken together, these features warrant the practical application of *Ec*PDH E1 for carboligation reactions with pyruvate and 2-oxobutyrate. While thermodynamically controlled self-reactions are 100% atom efficient, large hydration constants render this approach economically and environmentally unviable for most non-aromatic aldehyde substrates. In addition, a lower level of stereocontrol was observed for the self-reaction of propionaldehyde with *Ec*PDH E1. Thermodynamically controlled reactions with ThDP dependent enzymes therefore not only deviate with respect to the maximum yields from their kinetically controlled analogues, but may also differ in the enantiomeric purity of the product.

## Figures and Tables

**Table 1:** Synthetic performance of *Ec*PDH E1 on preparative scale. *Ec*PDH E1 consistently afforded the acyloin products (Figures S24–S33) in good enantiomeric purity from both aliphatic and hydroxyaldehyde substrates (Figures S6–S16). Conditions: 0.5 mg/mL WT *Ec*PDH E1, 100–120 mM of each substrate loading, 20 mM KPi, pH 7.0, 24 h, room temperature. Conversions were determined with respect to 1.0 equivalents of the ketoacid substrate.

Product	Conversion (%)	Isolated Yield (%)	*ee* (%)
**3a**	64	5	93
**3b**	44	43	93
**5b**	78	68	95
**7a**	99	57	95
**9a**	69	22	93

**Table 2:** Comparison of apparent kinetic parameters for different enzymes and substrates.

Substrate	Enzyme	Variant	U/mg	K_M_	k_cat_	k_cat_/K_M_	Ref.
				(mM)	(s^−1^)	(s^−1^mM^−1^)	
Pyruvate	TK_GST_	H102L/H474S	n.a. [g]	16.6 [a]	0.17 [a]	0.01 [a]	[40]
	*Ec*DXS	WT	n.a. [g]	3.3 [a]	0.50 [a]	0.15 [a]	[40]
	*Ec*E1	WT	2.95 [a]	4.2 [a]	10.3 [a]	2.45 [a]	t.w. [f]
HPA	TK_GST_	WT	n.a. [g]	2.3 [a]	12.7 [a]	5.50 [a]	[40]
	*Sc*TK	R528Q/S527T	0.44 [a]	53 [a]	n.a. [g]	n.a. [g]	[12]
	*Ec*E1	WT	0.24 [a]	100 [a]	0.83 [a]	0.008 [a]	t.w. [f]
2-oxobutyrate	TK_GST_	H102L/H474S	0.006 [e]	3.3 [a]	0.16 [a]	0.048 [a]	[40,41]
	*Ec*E1	WT	2.72 [a]	1.09 [a]	9.46 [a]	8.68 [a]	t.w. [f]
	*Ec*E1	WT	2.33 [e]				t.w. [f]
glycolaldehyde	*Ec*TK	H461S	3.14 [d]	n.a. [g]	n.a. [g]	n.a. [g]	[11]
	TK_GST_	L191I	4.07 [d]	n.a. [g]	n.a. [g]	n.a. [g]	[15]
	*Ec*E1	WT	3.09 [b]	25.2 [b]	10.7 [b]	0.42 [b]	t.w. [f]
propanal	TK_GST_	L191I/D470L	1.48 [d]	n.a. [g]	n.a. [g]	n.a. [g]	[15]
	TK_GST_	H102L/H474S/ F435I	0.007 [c]	n.a. [g]	n.a. [g]	n.a. [g]	[41]
	*Ec*E1	WT	2.33 [c]	42.4 [c]	8.10 [c]	0.19 [c]	t.w. [f]
isobutanal	*Sc*TK	D477E	n.a. [g]	66 [d]	0.6 [d]	0.009 [d]	[5]
	*Ec*E1	WT	0.78 [b]	69.4 [b]	2.70 [b]	0.039 [b]	t.w. [f]

In combination with: [a] glycolaldehyde, [b] pyruvate, [c] 2-oxobutyrate, [d] hydroxypyruvate, [e] propanal. [f] t.w. = this work. [g] n.a. = data not available.

## Supplementary Materials

The following are available online athttps://www.mdpi.com/1422-0067/21/22/8641/s1.

## Abbreviations

ThDP	Thiamine diphosphate
TK	Transketolase
*Ec*PDH E1	Pyruvate dehydrogenase E1 subunit from *Escherichia coli*
PDHc	Pyruvate dehydrogenase complex
DXS	1-deoxy-D-xylulose-5-phosphate synthase
MenD	2-succinyl-5-enolpyruvyl-6-hydroxy-3-cyclohexene-1-carboxylate synthase
BAL	Benzaldehyde lyase
DCPIP	2,6-dichloroindophenol
HPA	Hydroxypyruvate
TEOA	Triethanolamine
TEED	Thiamine Enzyme Engineering Database
