# Supplementary material for: Assessing the Thiamine Diphosphate Dependent Pyruvate Dehydrogenase E1 Subunit for Carboligation Reactions with Aliphatic Ketoacids
Source: Int J Mol Sci. 2020 Nov 16;21(22):8641. doi: 10.3390/ijms21228641 (PMC7696235; doi:10.3390/ijms21228641)
Supplement: Supplementary file 1 [file ijms-21-08641-s001.zip › ijms-980340-supplementary.docx]

**Supporting Information**

Assessing the Thiamine Diphosphate Dependent Pyruvate Dehydrogenase E1 Subunit for Carboligation Reactions with Aliphatic Ketoacids

Stefan R. Marsden, Duncan G.G. McMillan and Ulf Hanefeld *

Biokatalyse, Afdeling Biotechnologie, Technische Universiteit Delft, Van der Maasweg 9, 2629HZ Delft, The Netherlands; S.R.Marsden@tudelft.nl (S.R.M.); D.G.G.McMillan@tudelft.nl (D.G.G.M.)

***** Correspondence: u.hanefeld@tudelft.nl

***Ec*PDH E1 gene and protein sequences 2**

**Chemicals 5**

**Molecular biology 6**

**Expression and purification 6**

**DCPIP assay 9**

**Preparative scale reactions 10**

**Time course for the conversion of racemic DL-glyceraldehyde 12**

**GC programs and GC/LC chromatograms 12**

**Structural comparison of TK_GST_ and EcPDH E1 16**

**Michaelis-Menten curves 17**

**NMR spectrums 20**

**Calibration lines 25**

**Supplementary references 28**

***Ec*PDH E1 native aceE gene sequence**

Derived from *Escherichia coli* K-12, substrain MG1655, gene accession number: P0AFG9

ATGTCAGAACGTTTCCCAAATGACGTGGATCCGATCGAAACTCGCGACTGGCTCCAGGCGATCGAATCGGTCATCCGTGAAGAAGGTGTTGAGCGTGCTCAGTATCTGATCGACCAACTGCTTGCTGAAGCCCGCAAAGGCGGTGTAAACGTAGCCGCAGGCACAGGTATCAGCAACTACATCAACACCATCCCCGTTGAAGAACAACCGGAGTATCCGGGTAATCTGGAACTGGAACGCCGTATTCGTTCAGCTATCCGCTGGAACGCCATCATGACGGTGCTGCGTGCGTCGAAAAAAGACCTCGAACTGGGCGGCCATATGGCGTCCTTCCAGTCTTCCGCAACCATTTATGATGTGTGCTTTAACCACTTCTTCCGTGCACGCAACGAGCAGGATGGCGGCGACCTGGTTTACTTCCAGGGCCACATCTCCCCGGGCGTGTACGCTCGTGCTTTCCTGGAAGGTCGTCTGACTCAGGAGCAGCTGGATAACTTCCGTCAGGAAGTTCACGGCAATGGCCTCTCTTCCTATCCGCACCCGAAACTGATGCCGGAATTCTGGCAGTTCCCGACCGTATCTATGGGTCTGGGTCCGATTGGTGCTATTTACCAGGCTAAATTCCTGAAATATCTGGAACACCGTGGCCTGAAAGATACCTCTAAACAAACCGTTTACGCGTTCCTCGGTGACGGTGAAATGGACGAACCGGAATCCAAAGGTGCGATCACCATCGCTACCCGTGAAAAACTGGATAACCTGGTCTTCGTTATCAACTGTAACCTGCAGCGTCTTGACGGCCCGGTCACCGGTAACGGCAAGATCATCAACGAACTGGAAGGCATCTTCGAAGGTGCTGGCTGGAACGTGATCAAAGTGATGTGGGGTAGCCGTTGGGATGAACTGCTGCGTAAGGATACCAGCGGTAAACTGATCCAGCTGATGAACGAAACCGTTGACGGCGACTACCAGACCTTCAAATCGAAAGATGGTGCGTACGTTCGTGAACACTTCTTCGGTAAATATCCTGAAACCGCAGCACTGGTTGCAGACTGGACTGACGAGCAGATCTGGGCACTGAACCGTGGTGGTCACGATCCGAAGAAAATCTACGCTGCATTCAAGAAAGCGCAGGAAACCAAAGGCAAAGCGACAGTAATCCTTGCTCATACCATTAAAGGTTACGGCATGGGCGACGCGGCTGAAGGTAAAAACATCGCGCACCAGGTTAAGAAAATGAACATGGACGGTGTGCGTCATATCCGCGACCGTTTCAATGTGCCGGTGTCTGATGCAGATATCGAAAAACTGCCGTACATCACCTTCCCGGAAGGTTCTGAAGAGCATACCTATCTGCACGCTCAGCGTCAGAAACTGCACGGTTATCTGCCAAGCCGTCAGCCGAACTTCACCGAGAAGCTTGAGCTGCCGAGCCTGCAAGACTTCGGCGCGCTGTTGGAAGAGCAGAGCAAAGAGATCTCTACCACTATCGCTTTCGTTCGTGCTCTGAACGTGATGCTGAAGAACAAGTCGATCAAAGATCGTCTGGTACCGATCATCGCCGACGAAGCGCGTACTTTCGGTATGGAAGGTCTGTTCCGTCAGATTGGTATTTACAGCCCGAACGGTCAGCAGTACACCCCGCAGGACCGCGAGCAGGTTGCTTACTATAAAGAAGACGAGAAAGGTCAGATTCTGCAGGAAGGGATCAACGAGCTGGGCGCAGGTTGTTCCTGGCTGGCAGCGGCGACCTCTTACAGCACCAACAATCTGCCGATGATCCCGTTCTACATCTATTACTCGATGTTCGGCTTCCAGCGTATTGGCGATCTGTGCTGGGCGGCTGGCGACCAGCAAGCGCGTGGCTTCCTGATCGGCGGTACTTCCGGTCGTACCACCCTGAACGGCGAAGGTCTGCAGCACGAAGATGGTCACAGCCACATTCAGTCGCTGACTATCCCGAACTGTATCTCTTACGACCCGGCTTACGCTTACGAAGTTGCTGTCATCATGCATGACGGTCTGGAGCGTATGTACGGTGAAAAACAAGAGAACGTTTACTACTACATCACTACGCTGAACGAAAACTACCACATGCCGGCAATGCCGGAAGGTGCTGAGGAAGGTATCCGTAAAGGTATCTACAAACTCGAAACTATTGAAGGTAGCAAAGGTAAAGTTCAGCTGCTCGGCTCCGGTTCTATCCTGCGTCACGTCCGTGAAGCAGCTGAGATCCTGGCGAAAGATTACGGCGTAGGTTCTGACGTTTATAGCGTGACCTCCTTCACCGAGCTGGCGCGTGATGGTCAGGATTGTGAACGCTGGAACATGCTGCACCCGCTGGAAACTCCGCGCGTTCCGTATATCGCTCAGGTGATGAACGACGCTCCGGCAGTGGCATCTACCGACTATATGAAACTGTTCGCTGAGCAGGTCCGTACTTACGTACCGGCTGACGACTACCGCGTACTGGGTACTGATGGCTTCGGTCGTTCCGACAGCCGTGAGAACCTGCGTCACCACTTCGAAGTTGATGCTTCTTATGTCGTGGTTGCGGCGCTGGGCGAACTGGCTAAACGTGGCGAAATCGATAAGAAAGTGGTTGCTGACGCAATCGCCAAATTCAACATCGATGCAGATAAAGTTAACCCGCGTCTGGCGTAA

**Native Protein sequence:**

MSERFPNDVDPIETRDWLQAIESVIREEGVERAQYLIDQLLAEARKGGVNVAAGTGISNYINTIPVEEQPEYPGNLELERRIRSAIRWNAIMTVLRASKKDLELGGHMASFQSSATIYDVCFNHFFRARNEQDGGDLVYFQGHISPGVYARAFLEGRLTQEQLDNFRQEVHGNGLSSYPHPKLMPEFWQFPTVSMGLGPIGAIYQAKFLKYLEHRGLKDTSKQTVYAFLGDGEMDEPESKGAITIATREKLDNLVFVINCNLQRLDGPVTGNGKIINELEGIFEGAGWNVIKVMWGSRWDELLRKDTSGKLIQLMNETVDGDYQTFKSKDGAYVREHFFGKYPETAALVADWTDEQIWALNRGGHDPKKIYAAFKKAQETKGKATVILAHTIKGYGMGDAAEGKNIAHQVKKMNMDGVRHIRDRFNVPVSDADIEKLPYITFPEGSEEHTYLHAQRQKLHGYLPSRQPNFTEKLELPSLQDFGALLEEQSKEISTTIAFVRALNVMLKNKSIKDRLVPIIADEARTFGMEGLFRQIGIYSPNGQQYTPQDREQVAYYKEDEKGQILQEGINELGAGCSWLAAATSYSTNNLPMIPFYIYYSMFGFQRIGDLCWAAGDQQARGFLIGGTSGRTTLNGEGLQHEDGHSHIQSLTIPNCISYDPAYAYEVAVIMHDGLERMYGEKQENVYYYITTLNENYHMPAMPEGAEEGIRKGIYKLETIEGSKGKVQLLGSGSILRHVREAAEILAKDYGVGSDVYSVTSFTELARDGQDCERWNMLHPLETPRVPYIAQVMNDAPAVASTDYMKLFAEQVRTYVPADDYRVLGTDGFGRSDSRENLRHHFEVDASYVVVAALGELAKRGEIDKKVVADAIAKFNIDADKVNPRLA

***N*-His_6_-*Ec*PDH E1 codon optimised gene sequence:**

ATGGGGGGTTCTCATCATCATCATCATCATGGTATGGCTAGCATGACTGGTGGACAGCAAATGGGTCGGGATCTGTACGACGATGACGATAAGGATCGATGGGGATCCGAGCTCGAGCCCATGGGCATGTCAGAACGTTTCCCAAATGACGTGGATCCGATCGAAACTCGCGACTGGCTCCAGGCGATCGAATCGGTCATCCGTGAAGAAGGTGTTGAGCGTGCTCAGTATCTGATCGACCAACTGCTTGCTGAAGCCCGCAAAGGCGGTGTAAACGTAGCCGCAGGCACAGGTATCAGCAACTACATCAACACCATCCCCGTTGAAGAACAACCGGAGTATCCGGGTAATCTGGAACTGGAACGCCGTATTCGTTCAGCTATCCGCTGGAACGCCATCATGACGGTGCTGCGTGCGTCGAAAAAAGACCTCGAACTGGGCGGCCATATGGCGTCCTTCCAGTCTTCCGCAACCATTTATGATGTGTGCTTTAACCACTTCTTCCGTGCACGCAACGAGCAGGATGGCGGCGACCTGGTTTACTTCCAGGGCCACATCTCCCCGGGCGTGTACGCTCGTGCTTTCCTGGAAGGTCGTCTGACTCAGGAGCAGCTGGATAACTTCCGTCAGGAAGTTCACGGCAATGGCCTCTCTTCCTATCCGCACCCGAAACTGATGCCGGAATTCTGGCAGTTCCCGACCGTATCTATGGGTCTGGGTCCGATTGGTGCTATTTACCAGGCTAAATTCCTGAAATATCTGGAACACCGTGGCCTGAAAGATACCTCTAAACAAACCGTTTACGCGTTCCTCGGTGACGGTGAAATGGACGAACCGGAATCCAAAGGTGCGATCACCATCGCTACCCGTGAAAAACTGGATAACCTGGTCTTCGTTATCAACTGTAACCTGCAGCGTCTTGACGGCCCGGTCACCGGTAACGGCAAGATCATCAACGAACTGGAAGGCATCTTCGAAGGTGCTGGCTGGAACGTGATCAAAGTGATGTGGGGTAGCCGTTGGGATGAACTGCTGCGTAAGGATACCAGCGGTAAACTGATCCAGCTGATGAACGAAACCGTTGACGGCGACTACCAGACCTTCAAATCGAAAGATGGTGCGTACGTTCGTGAACACTTCTTCGGTAAATATCCTGAAACCGCAGCACTGGTTGCAGACTGGACTGACGAGCAGATCTGGGCACTGAACCGTGGTGGTCACGATCCGAAGAAAATCTACGCTGCATTCAAGAAAGCGCAGGAAACCAAAGGCAAAGCGACAGTAATCCTTGCTCATACCATTAAAGGTTACGGCATGGGCGACGCGGCTGAAGGTAAAAACATCGCGCACCAGGTTAAGAAAATGAACATGGACGGTGTGCGTCATATCCGCGACCGTTTCAATGTGCCGGTGTCTGATGCAGATATCGAAAAACTGCCGTACATCACCTTCCCGGAAGGTTCTGAAGAGCATACCTATCTGCACGCTCAGCGTCAGAAACTGCACGGTTATCTGCCAAGCCGTCAGCCGAACTTCACCGAGAAGCTTGAGCTGCCGAGCCTGCAAGACTTCGGCGCGCTGTTGGAAGAGCAGAGCAAAGAGATCTCTACCACTATCGCTTTCGTTCGTGCTCTGAACGTGATGCTGAAGAACAAGTCGATCAAAGATCGTCTGGCACCGATCATCGCCGACGAAGCGCGTACTTTCGGTATGGAAGGTCTGTTCCGTCAGATTGGTATTTACAGCCCGAACGGTCAGCAGTACACCCCGCAGGACCGCGAGCAGGTTGCTTACTATAAAGAAGACGAGAAAGGTCAGATTCTGCAGGAAGGGATCAACGAGCTGGGCGCAGGTTGTTCCTGGCTGGCAGCGGCGACCTCTTACAGCACCAACAATCTGCCGATGATCCCGTTCTACATCTATTACTCGATGTTCGGCTTCCAGCGTATTGGCGATCTGTGCTGGGCGGCTGGCGACCAGCAAGCGCGTGGCTTCCTGATCGGCGGTACTTCCGGTCGTACCACCCTGAACGGCGAAGGTCTGCAGCACGAAGATGGTCACAGCCACATTCAGTCGCTGACTATCCCGAACTGTATCTCTTACGACCCGGCTTACGCTTACGAAGTTGCTGTCATCATGCATGACGGTCTGGAGCGTATGTACGGTGAAAAACAAGAGAACGTTTACTACTACATCACTACGCTGAACGAAAACTACCACATGCCGGCAATGCCGGAAGGTGCTGAGGAAGGTATCCGTAAAGGTATCTACAAACTCGAAACTATTGAAGGTAGCAAAGGTAAAGTTCAGCTGCTCGGCTCCGGTTCTATCCTGCGTCACGTCCGTGAAGCAGCTGAGATCCTGGCGAAAGATTACGGCGTAGGTTCTGACGTTTATAGCGTGACCTCCTTCACCGAGCTGGCGCGTGATGGTCAGGATTGTGAACGCTGGAACATGCTGCACCCGCTGGAAACTCCGCGCGTTCCGTATATCGCTCAGGTGATGAACGACGCTCCGGCAGTGGCATCTACCGACTATATGAAACTGTTCGCTGAGCAGGTCCGTACTTACGTACCGGCTGACGACTACCGCGTACTGGGTACTGATGGCTTCGGTCGTTCCGACAGCCGTGAGAACCTGCGTCACCACTTCGAAGTTGATGCTTCTTATGTCGTGGTTGCGGCGCTGGGCGAACTGGCTAAACGTGGCGAAATCGATAAGAAAGTGGTTGCTGACGCAATCGCCAAATTCAACATCGATGCAGATAAAGTTAACCCGCGTCTGGCGTAA

***N*-His_6_ Protein sequence:** A linker was introduced between the *N*-His_6_-tag and the target protein by the cloning strategy of the aceE gene into the pBAD/HisA plasmid.
MW = 104.3 kDa (Monomer)

MGGSHHHHHHGMASMTGGQQMGRDLYDDDDKDRWGSELEPMGMSERFPNDVDPIETRDWLQAIESVIREEGVERAQYLIDQLLAEARKGGVNVAAGTGISNYINTIPVEEQPEYPGNLELERRIRSAIRWNAIMTVLRASKKDLELGGHMASFQSSATIYDVCFNHFFRARNEQDGGDLVYFQGHISPGVYARAFLEGRLTQEQLDNFRQEVHGNGLSSYPHPKLMPEFWQFPTVSMGLGPIGAIYQAKFLKYLEHRGLKDTSKQTVYAFLGDGEMDEPESKGAITIATREKLDNLVFVINCNLQRLDGPVTGNGKIINELEGIFEGAGWNVIKVMWGSRWDELLRKDTSGKLIQLMNETVDGDYQTFKSKDGAYVREHFFGKYPETAALVADWTDEQIWALNRGGHDPKKIYAAFKKAQETKGKATVILAHTIKGYGMGDAAEGKNIAHQVKKMNMDGVRHIRDRFNVPVSDADIEKLPYITFPEGSEEHTYLHAQRQKLHGYLPSRQPNFTEKLELPSLQDFGALLEEQSKEISTTIAFVRALNVMLKNKSIKDRLAPIIADEARTFGMEGLFRQIGIYSPNGQQYTPQDREQVAYYKEDEKGQILQEGINELGAGCSWLAAATSYSTNNLPMIPFYIYYSMFGFQRIGDLCWAAGDQQARGFLIGGTSGRTTLNGEGLQHEDGHSHIQSLTIPNCISYDPAYAYEVAVIMHDGLERMYGEKQENVYYYITTLNENYHMPAMPEGAEEGIRKGIYKLETIEGSKGKVQLLGSGSILRHVREAAEILAKDYGVGSDVYSVTSFTELARDGQDCERWNMLHPLETPRVPYIAQVMNDAPAVASTDYMKLFAEQVRTYVPADDYRVLGTDGFGRSDSRENLRHHFEVDASYVVVAALGELAKRGEIDKKVVADAIAKFNIDADKVNPRLA

**Chemicals:** chemicals were generally bought in the highest purity commercially available and microbiological work was carried out under sterile conditions using autoclaved materials. Lithium β-hydroxypyruvate (Sigma Aldrich, >97%), sodium pyruvate (Sigma Aldrich, >99%), sodium 2-oxobutyrate (Sigma Aldrich, >98%), glycolaldehyde (Sigma Aldrich, dimer, crystalline), DL-glyceraldehyde (Sigma Aldrich, crystalline, ≥90%), propionaldehyde (Sigma Aldrich, freshly distilled), butyraldehyde (Sigma Aldrich, freshly distilled), isobutyraldehyde (Sigma Aldrich, freshly distilled), L-erythrulose (Sigma Aldrich, 85%), 2,6-dichloroindophenol sodium salt hydrate (Fluka, 97%), thiamine diphosphate (Sigma Aldrich, >95%), magnesium chloride hexahydrate (J.T. Baker), potassium dihydrogen phosphate (Sigma Aldrich, 99.5%), dipotassium hydrogen phosphate (Sigma Aldrich, ≥98%), potassium hydroxide (Acros Organics, 85%), sodium chloride (Sigma Aldrich, ≥99.5%), yeast extract (BD Becton Dickinson), tryptone (BD Bacto Peptone, BD Biosciences), L(+)-arabinose (Sigma Aldrich, ≥99%), ampicillin sodium salt (Sigma Aldrich), imidazole (Sigma Aldrich, ≥99%), lysozyme (Sigma Aldrich, from chicken egg white, lyophilized powder, ≥90%, ≥40000 U/mg, EC 3.2.1.17) deoxyribonuclease I (Sigma Aldrich, from bovine pancreas, lyophilized powder, ≥85%, ≥400 Kunitz U/mg, EC 3.1.21.1), cOmplete EDTA-free protease inhibitor cocktail (Sigma Aldrich), Ni-Sepharose 6 Fast Flow resin (GE Healthcare, 6% cross-linked agarose), Pierce BCA protein assay kit (Thermo Fisher Scientific). WT *Ec*PDH E1 and H640S/Y177S *Ec*PDH E1 were synthesised by BaseClear B.V. (The Netherlands).

**Restriction digestion:** Plasmid DNA (2 µg) was digested with KpnI and XhoI (1 µL/50 µL) in CutSmart buffer (New England Biolabs, 37°C, 1 h). Purple loading dye was added (10 µL) and the samples were analysed by gel electrophoresis (1% agarose in TAE buffer, 1x SYBR safe stain, 100 V, 400 mA, 50 min.). DNA fragments were extracted from the corresponding gel slabs using the Qiagen gel extraction kit and were subsequently used for ligations.

**Ligation:** The restricted DNA fragments were mixed at a 1:1.9 ratio of insert:vector (pBAD/His A) in T4 Ligase buffer (19 µL) and T4 DNA ligase was added (1 µL). The mixtures were incubated at room temperature (30 min) and subsequently used for transformations.

**Transformation:** 50 µL of CaCl_2_ competent cells were thawed on ice (30 min.) before the addition of plasmid DNA (5 µL), followed by further incubation on ice (30 min.). The cells were subjected to a heat shock (42°C, 60 sec.) and subsequently incubated on ice (2 min.). LB medium was added (500 µL) and the cells were incubated (37°C, 1 h) after which they were plated on selective, solid medium (LB agar containing 100 µg/mL ampicillin).

**PCR mutagenesis:** Q5 hot start high-fidelity master mix was mixed with the plasmid template (10 ng), forward and reverse primers (10 µM each) and filled up to a total volume of 25 µL. PCR protocol: 98°C (30 s) initial denaturation. Recycle sequence (35x): 98°C (10 s), 69°C (30 s), 72°C (7 min). The template plasmid was subsequently digested with DpnI (37°C, 2h), after which the enzyme was inactivated (80°C, 20 min). Competent cells were subsequently transformed with the crude PCR product using the above protocol.

Table S1: Primer sequences used for the PCR mutagenesis (5’ to 3’). Mutant H640S was created by reversing the mutation S177Y in the synthetic double mutant H640S/Y177S.

| **Primer** | **Sequence** | **Template** |
| --- | --- | --- |
| Y177S_FW | CCTCTCTTCCTCTCCGCACCC | WT |
| Y177S_REV | GGGTGCGGAGAGGAAGAGAGG | WT |
| S177Y_FW | CCTCTCTTCCTATCCGCACCC | H640S/Y177S |
| S177Y_REV | GGGTGCGGATAGGAAGAGAGG | H640S/Y177S |

**Batch fermentation expressing *Ec*PDH E1:** The fermenter was charged with medium (2 L terrific broth, 12 g/L tryptone, 24 g/L yeast extract, 4 mL/L glycerol, 2.2 g/L KH_2_PO_4_, 9.4 g/L K_2_HPO_4_, pH = 7.0), autoclaved at 121°C and sterilised anti-foam, acid (3M H_3_PO_4_) and base (28% NH_4_OH) were subsequently attached to the system. Filter sterilised (0.2 µm) ampicillin was added at a final concentration of 100 µg/mL. A pre-culture of *E. coli* top 10 containing the aceE gene in the pBAD/HisA plasmid was grown overnight (50 mL LB in a 500 mL flat bottom Erlenmeyer flask, 100 µg/mL ampicillin, 37°C, 180 rpm) of which 25 mL were used as inoculum (1:80 dilution). The fermenter was operated at constant conditions with a stirring speed of 500 rpm (0-6h, then 750 rpm until the end), aeration of 0.5 L/min (corresponding to 0.25 v/v/min), 0.2 bar head pressure, 37°C, and the pH was kept constant at 7.0. Gene expression was induced at OD_600_ = 0.6 with filter sterilised L-arabinose (0.02% w/v final concentration) after which the fermenter was operated under the aforementioned conditions until substrate depletion was observed by an increase in dO_2_. The cells were harvested by centrifugation (10’000 rpm, 15 min, 4°C), washed with buffer (30 mL 20 mM KH_2_PO_4_, pH = 7.0) to afford 31 g of wet cell pellet which was stored at -80°C.

Figure S1: Dissolved oxygen (dO_2_) profile during the batch fermentation of *Ec*PDH E1. After 6 hours, the stirring was increased from 600 rpm to 750 rpm, leading to a spike in dissolved oxygen. After 9 hours, readily accessible carbon sources presumably became depleted and adjustment of metabolism lead to a temporary phase of low metabolic activity. After 16 hours, all nutrients were consumed, and the fermentation was stopped.

**Affinity purification:** The cell pellet was thawed on ice and suspended in lysis buffer at 5 mL per gram of wet cell weight (20 mM KPi, 0.5 M NaCl, 20 mM imidazole, 1 tablet cOmplete EDTA-free protease inhibitor cocktail, 1 mg/mL lysozyme, 5 mg deoxy ribonuclease I). The cells were disrupted with a Constant Systems cell disrupter during three successive rounds (1.8 kbar) and the cell debris was removed by centrifugation (18’000 rcf, 30 min. 4°C). The supernatant was filtered (0.45 µm pore size) and the obtained cell-free extract was purified by immobilised metal affinity chromatography using a custom packed Ni-sepharose 6 fast flow column. The collected fractions were analysed by SDS-PAGE and the combined fractions were concentrated using Amicon ultrafiltration tubes (30 kDa molecular weight cut off, 5’000 rcf, 4°C). The combined fractions (~50 mL) were dialysed two times (20 mM KH_2_PO_4_ buffer, pH = 7.0, 1:40 dilution, 8h – overnight, 3’500 Da molecular weight cut off). The protein concentration was determined with the BCA assay and aliquots of 1 mL at 5 mg/mL were flash frozen in liquid nitrogen. Aliquots were stored at -20°C until use.


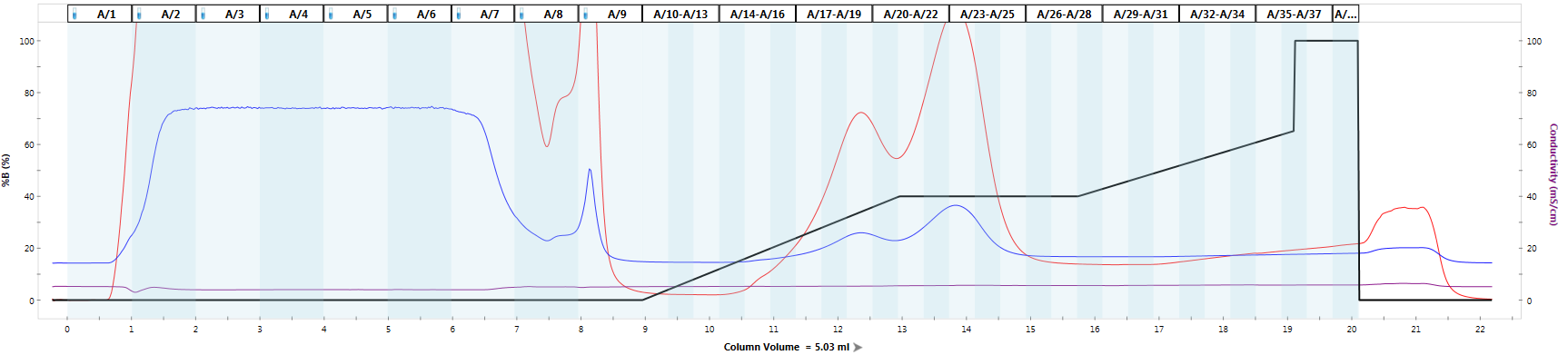


Figure S2: Purification of the *Ec*PDH E1 enzyme by immobilized metal affinity chromatography. Elution was carried out with an imidazole gradient (100% = 500 mM). Separate elution of the monomeric (one His_6_-tag) and dimeric forms (two His_6_-tags) were observed, due to the different binding strengths.


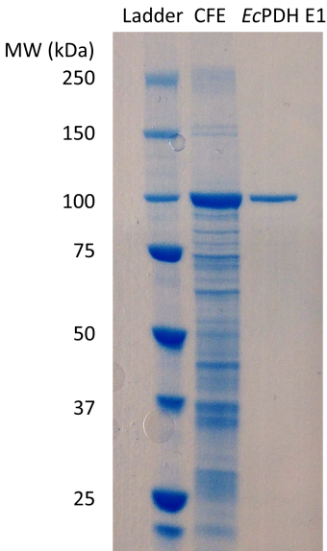


Figure S3: SDS-PAGE analysis of purified *Ec*PDH E1. The cell-free extract (CFE) shows good overexpression of the target protein (104.3 kDa). Combination of the two peaks from the elution profile shown in Figure S2 leads to a single band during SDS-PAGE analysis, corresponding to the molecular weight of the monomer. This suggests that the enzyme elutes in its monomeric and dimeric form during metal affinity purification, similar to the elution profile of transketolase from *Saccharomyces cerevisiae* [5].


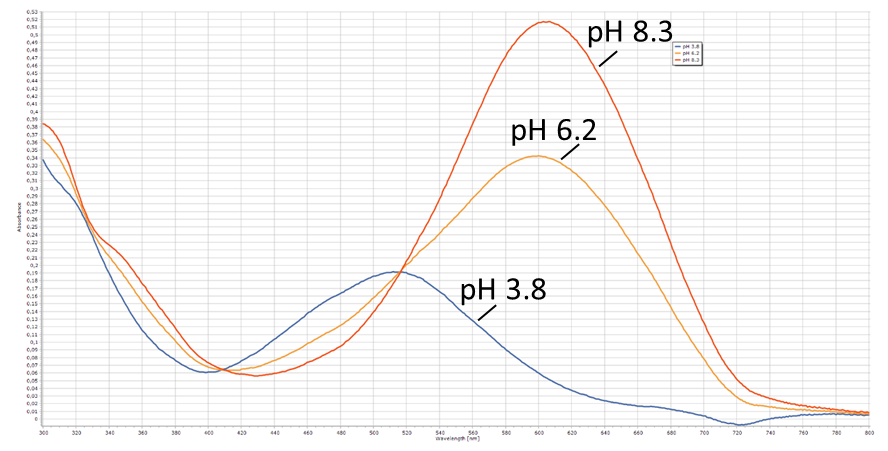


Figure S4: Absorbance spectra of 2,6-dichloroindophenol at pH 3.8, 6.2 and 8.3. The isosbestic point was determined at 517 nm and was subsequently used for the pH optimisation by the DCPIP assay.

**Scheme S1:** 2,6-Dichloroindophenol (DCPIP) assay to measure *Ec*PDH E1 activity [52]. DCPIP reacts with the carbanion intermediate, that is formed between pyruvate and the ThDP cofactor (the pyrimidine ring and the pyrophosphate groups are abbreviated as R’ and R’’). During this reaction, DCPIP is reduced and a permanent acetyl adduct is formed with the ThDP cofactor.

**Preparative scale reactions:**

**4-deoxy-erythrulose 3a:**



*Ec*PDH E1 (20 mg) was incubated with ThDP (0.2 mM) and MgCl_2_ (2 mM) in potassium phosphate buffer (100 mL, 20 mM, pH 7.0) for 5 min, after which sodium pyruvate (1.1 g, 100 mM, 1.05 eq.) and glycolaldehyde (570 mg, 9.5 mmol, 1.0 eq.) were added. The reaction was carried out overnight at room temperature, after which the product was extracted with MTBE (3x 150 mL). The organic phase was dried over MgSO_4_ and the solvent was removed *in vacuo* to afford the product as a pale oil (46 mg, 4.5% yield). A low extraction efficiency was responsible for the low yield, which could be improved by first removing the water *in vacuo*.

^1^H-NMR (400 MHz, CDCl_3_, 298K, δ/ppm), Figure S24: 4.23 (t, ^3^J_HH_ = 3.2 Hz, 1H, H2), 3.97-3.93 (dd, ^2^J_HH_ = 12.4 Hz, ^3^J_HH_ = 3.2 Hz, 1H, H1a), 3.91-3.87 (dd, ^2^J_HH_ = 12.4 Hz, ^3^J_HH_ = 3.2 Hz, 1H, H1b), 3.26 (s, broad, 2H, OH1, OH2), 2.26 (s, 3H, H4). ^13^C-NMR (100 MHz, CDCl_3_, 298K, δ/ppm), Figure S25: 208.6 (C3), 78.1 (C2), 63.3 (C1), 25.6 (C4), in agreement with literature [40]. GC: 11.7 min (*R*), 12.0 min (*S*), 92.7% *ee*, *S*-selective, Figure S7. HPLC: 10.2 min, Figure S6.

**(*S*)-1,2-dihydroxypentan-3-one 3b:**



 *Ec*PDH E1 (25 mg) was incubated with ThDP (0.2 mM) and MgCl_2_ (2 mM) in potassium phosphate buffer (50 mL, 20 mM, pH 7.0) for 5 min, after which sodium 2-oxobutyrate (620 mg, 100 mM, 1.0 eq.) and glycolaldehyde (360 mg, 120 mM, 1.2 eq.) were added. The reaction was carried out overnight at room temperature, after which the water was evaporated. The solid residue was dissolved in MTBE, dried over MgSO_4_ and the solvent was removed *in vacuo* to afford the product as a colourless oil (254 mg, 43% yield).

^1^H-NMR (400 MHz, CDCl_3_, 298K, δ/ppm), Figure S26: 4.23 (t, ^3^J_HH_ = 3.3 Hz, 1H, H2), 3.96 – 3.92 (dd, ^2^J_HH_ = 12.1 Hz, ^3^J_HH_ = 3.3 Hz, 1H, H1a), 3.89 – 3.85 (dd, ^2^J_HH_ = 12.1 Hz, ^3^J_HH_ = 3.3 Hz, 1H, H1b), 2.56 (m, 2H, H4), 1.12 (t, ^3^J_HH_ = 7.3 Hz, 3H, H5). ^13^C-NMR (100 MHz, CDCl_3_, 298K, δ/ppm), Figure S27: 211.4 (C3), 77.7 (C2), 63.5 (C1), 31.5 (C4), 7.1 (C5), in agreement with literature [40]. GC: 10.8 min (*R*), 11.1 min (*S*), 93.0% *ee*, *S*-selective, Figure S9. HPLC: 13.8 min, Figure S8.

**4-hydroxyhexan-3-one 5b:**



*Ec*PDH E1 (25 mg) was incubated with ThDP (0.2 mM) and MgCl_2_ (2 mM) in potassium phosphate buffer (50 mL, 20 mM, pH 7.0) for 5 min, after which sodium 2-oxobutyrate (150 mg, 120 mM, 1.2 eq.) and propionaldehyde (72 µL, 100 mM, 1.0 eq.) were added. The reaction was carried out overnight at room temperature, after which the product was extracted with MTBE (3x 150 mL). The organic phase was dried over MgSO_4_ and the solvent was removed *in vacuo* to afford the product as a colourless oil (79 mg, 68% yield).

^1^H-NMR (400 MHz, CDCl_3_, 298K, δ/ppm), Figure S28: 4.17 (dd, ^3^J_HH_ = 6.8 Hz, ^3^J_HH_ = 4.1 Hz, 1H, H4), 3.48 (s, broad, 1H, OH4), 2.66 – 2.36 (m, 2H, H2), 1.90 (dqd, ^2^J_HH_ = 14.0 Hz, ^3^J_HH_ = 7.5 Hz, ^3^J_HH_ = 4.0 Hz, 1H, H5a), 1.62 (dq, ^2^J_HH_ = 14.1 Hz, ^3^J_HH_ = 7.1 Hz, 1H, H5b), 1.12 (t, ^3^J_HH_ = 7.3 Hz, 3H, H1), 0.94 (t, ^3^J_HH_ = 7.4 Hz, 3H, H6). ^13^C-NMR (100 MHz, CDCl_3_, 298K, δ/ppm), Figure S29: 214.3 (C3), 77.2 (C4), 30.4 (C2), 26.4 (C5), 9.5 (C6), 7.3 (C1), in agreement with literature [58]. GC: 9.1 min. (*R*), 9.4 min. (*S*), 94.7% *ee*, *S*-selective, Figure S11. HPLC: 21.3 min, Figure S10.

**Thermodynamically controlled self-reaction of propionaldehyde to afford 5b:**



*Ec*PDH E1 (20 mg) was incubated with ThDP (0.2 mM) and MgCl_2_ (2 mM) in potassium phosphate buffer (10 mL, 20 mM, pH 7.5) for 5 min, after which propionaldehyde (36 µL, 50 mM) was added. Progress of the reaction was followed quantitatively by HPLC. Equilibrium conditions were demonstrated by the addition of extra enzyme, no change was observed. The product was extracted with MTBE, dried over MgSO_4_ and the enantiomeric excess was determined to be 67% by GC, Figure S12. Analytical scale reactions were carried out at 0.5 mg/mL enzyme loading.

**3-hydroxyhexan-2-one 7a:**



*Ec*PDH E1 (2 mg) was incubated with ThDP (0.2 mM) and MgCl_2_ (2 mM) in potassium phosphate buffer (4 mL, 20 mM, pH 7.0) for 5 min, after which sodium pyruvate (66 mg, 150 mM, 1.5 eq.) and butyraldehyde (36 µL, 100 mM, 1.0 eq.) were added. The reaction was carried out overnight at room temperature, after which it was extracted with MTBE (3x 15 mL). The organic phase was dried over MgSO_4_, and the solvent was removed *in vacuo* to afford the product as a colourless oil (26.4 mg, 57% yield).

^1^H-NMR (400 MHz, CDCl_3_, 298K, δ/ppm), Figure S30: 4.19 (m, 1H, H3), 3.44 (s, 1H, OH), 2.20 (s, 3H, H1), 1.82 (m, 1H, H4_a_), 1.55 (m, 1H, H4_b_), 1.51-1.36 (m, 2H, H5), 0.96 (t, ^3^J_HH_ = 7.1 Hz, 3H, H6). ^13^C-NMR (100 MHz, CDCl_3_, 298K, δ/ppm), Figure S31: 210.2 (C2), 76.8 (C3), 35.8 (C4), 25.3 (C1), 18.2 (C5), 14.0 (C6), in agreement with literature [58]. GC: 7.4 min (*R*), 7.8 min (*S*), 95.1% *ee, S*-selective, Figure S14. HPLC: 20.0 min, Figure S13.

Absolute configurations were determined for compounds **5b** and **7a** by comparison of GC traces with published literature references. [58] The absolute configuration of compounds **3ab** and **9a** were deduced from the notion, that the (*S*)-configured enantiomers interact more strongly with the column material.

**3-hydroxy-4-methylpentan-2-one 9a:**

*

Ec*PDH E1 (5 mg) was incubated with ThDP (0.2 mM) and MgCl_2_ (2 mM) in potassium phosphate buffer (10 mL, 20 mM, pH 7.5) for 5 min, after which isobutyraldehyde (91 µL, 100 mM, 1.0 eq.) and sodium pyruvate (132 mg, 120 mM, 1.2 eq.) were added. The reaction was carried out overnight at room temperature, after which the product was extracted with MTBE (3x 50 mL). The solvent was dried over MgSO_4_ and removed *in vacuo* to afford the product as a colourless oil (25 mg, 22% yield).

^1^H-NMR (400 MHz, CDCl_3_, 298K, δ/ppm), Figure S32: 4.07 (d, ^3^J_HH_ = 2.2 Hz, 1H, H3), 3.83 (s, 1H, OH), 2.18 (s, 3H, H1), 2.15 (m, 1H, H4), 1.11 (d, ^3^J_HH_ = 6.9 Hz, 3H, H5_a_), 0.71 (d, ^3^J_HH_ = 6.8 Hz, 3H, H5_b_). ^13^C-NMR (100 MHz, CDCl_3_, 298K, δ/ppm), Figure S33: 210.0 (C2), 81.3 (C3), 31.2 (C4), 25.6 (C1), 20.1 (C5_a_), 14.8 (C5_b_). GC: 9.1 min. (*R*), 9.6 min. (*S*), 92.5% *ee*, *S*-selective, Figure S16. HPLC: 19.4 min, Figure S15.


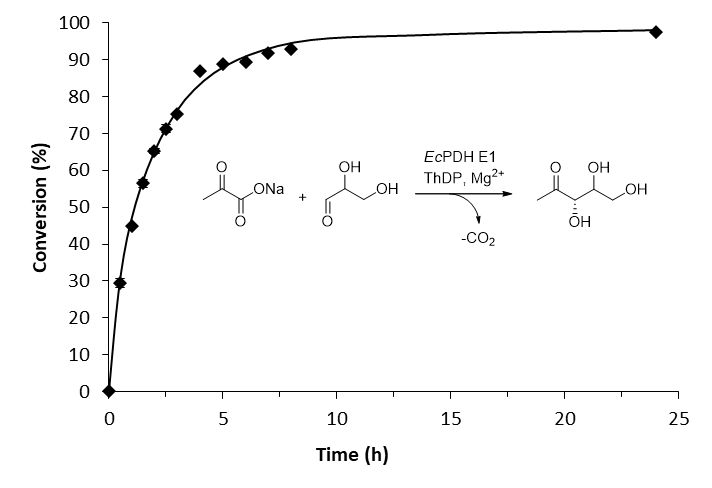


Figure S5: Time course of the *Ec*PDH E1 catalysed conversion of racemic DL-glyceraldehyde and sodium pyruvate (0.1 mg/mL *Ec*PDH E1, 0.2 mM ThDP, 2 mM MgCl_2_, 50 mM sodium pyruvate, 50 mM DL-glyceraldehyde, 20 mM KPi, pH 7.5, *n =* 3). Complete conversion indicated no stereopreference regarding the configuration α-hydroxyaldehyde substrates.

**GC and HPLC chromatograms**

Table S2: GC temperature programs used for the chiral separation of α-hydroxyketones and retention times on HPLC and chiral GC.

| Program | Compound | GC  (*R*) (min) | GC  (*S*) (min) | HPLC  (min) |
| --- | --- | --- | --- | --- |
| 70/4/15/140/5/15/225/2 | 4-deoxyerythrulose **3a** | 11.7 | 12.0 | 10.2 |
| 70/2/15/150/5/15/225/2 | 1,2-dihydroxypentan-3-one **3b** | 10.8 | 11.1 | 13.8 |
| 70/4/15/130/5/15/225/2 | 4-hydroxyhexan-3-one **5b** | 9.1 | 9.4 | 21.3 |
| 70/2/15/130/5/15/225/2 | 3-hydroxyhexan-2-one **7a** | 7.4 | 7.9 | 20.0 |
| 70/2/15/150/5/15/225/2 | 3-hydroxy-4-methylpentan-2-one **9a** | 9.1 | 9.6 | 19.4 |


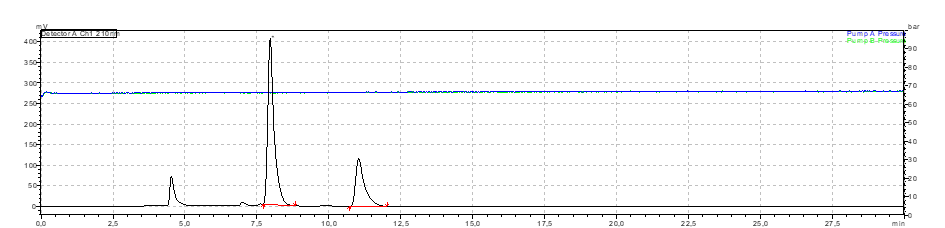


Figure S6: RP-HPLC chromatogram of 4-deoxy-L-erythrulose 3a (10.2 min) containing residual sodium pyruvate (7.9 min).


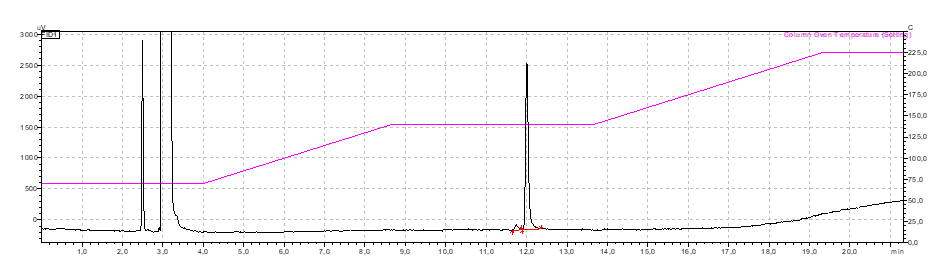


Figure S7: Chiral separation of enantiomers of 4-deoxy-L-erythrulose 3a by GC. 11.7 min (*R*), 12.0 min (*S*), 92.7% *ee*.


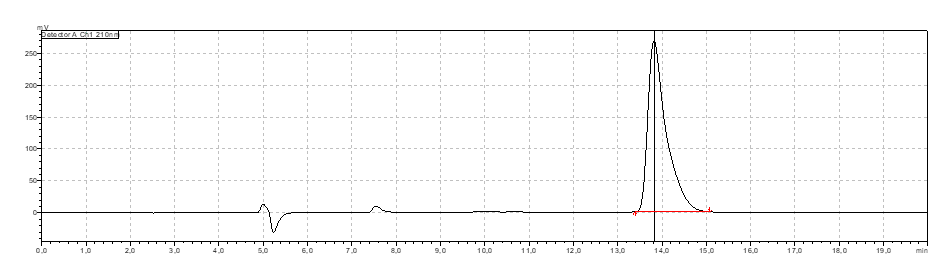


Figure S8: RP-HPLC chromatogram of (*S*)-1,2-dihydroxypentan-3-one 3b. Retention time: 13.8 min.


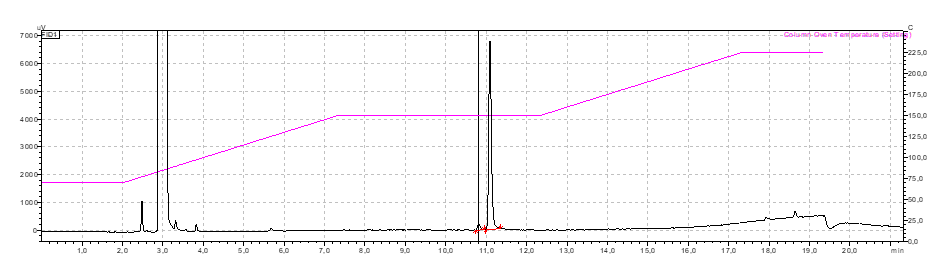


Figure S9: Chiral separation of enantiomers of 1,2-dihydroxypentan-3-one 3b by GC. 10.8 min (*R*), 11.1 min (*S*), 93% *ee*.


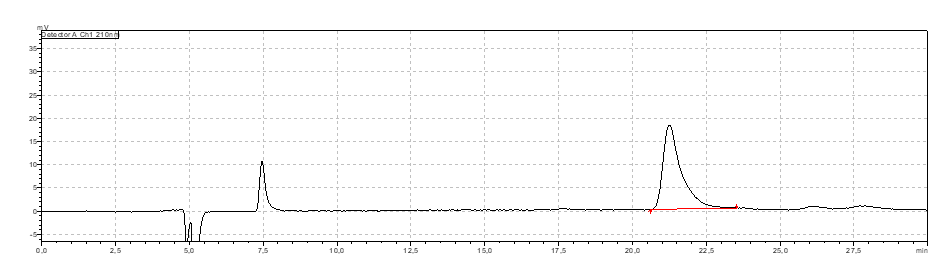


Figure S10: RP-HPLC chromatogram of 4-hydroxyhexan-3-one 5b. Retention time: 21.3 min.


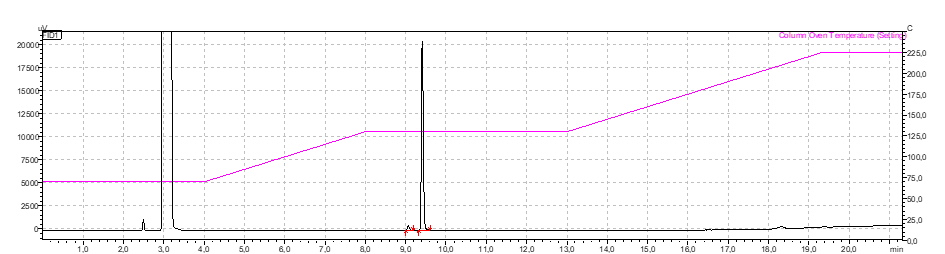


Figure S11: Chiral separation of enantiomers of 4-hydroxyhexan-3-one 5b by GC. 9.1 min (*R*), 9.4 min (*S*), 94.7% *ee*. The product was obtained by the decarboxylative coupling of 2-oxobutyrate and propionaldehyde under kinetically controlled conditions.


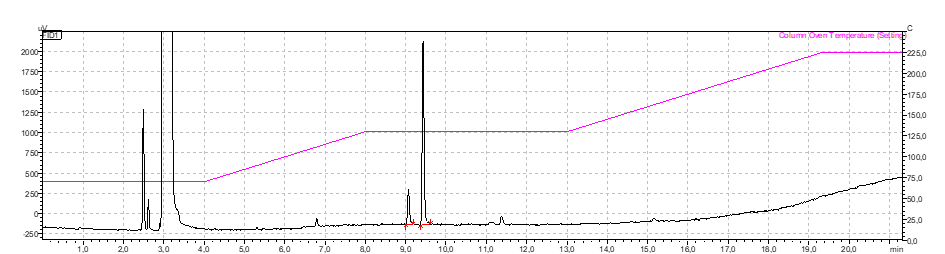


Figure S12: Chiral separation of enantiomers of 4-hydroxyhexan-3-one 5b by GC. 9.1 min (*R*), 9.4 min (*S*), 67.2% *ee*. The product was obtained *via* the self-reaction of propionaldehyde under thermodynamically controlled conditions.


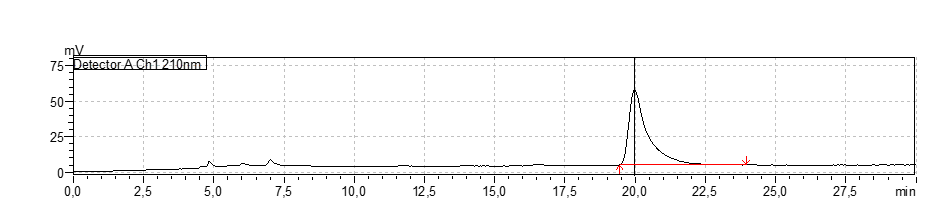


Figure S13: RP-HPLC chromatogram of (*3S*)-hydroxyhexan-2-one 7a. Retention time: 20.0 min.


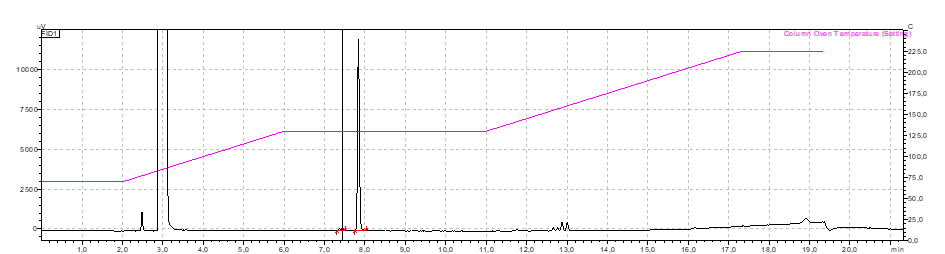


Figure S14: Chiral separation of enantiomers of 3-dihydroxyhexan-2-one 7a by GC. 7.4 min (*R*), 7.8 min (*S*), 95.1% *ee*.


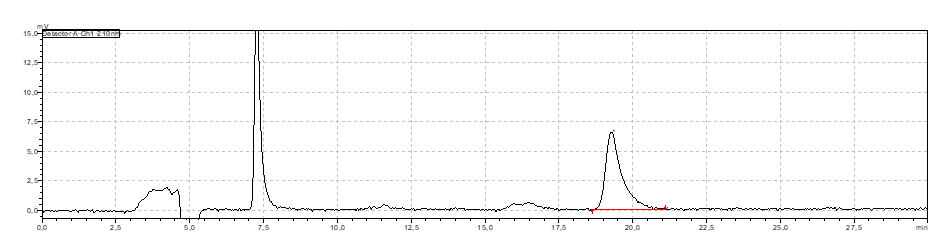


Figure S15: RP-HPLC chromatogram of (*3S*)-hydroxy-4-methylpentan-2-one 9a. Retention time: 19.4 min.


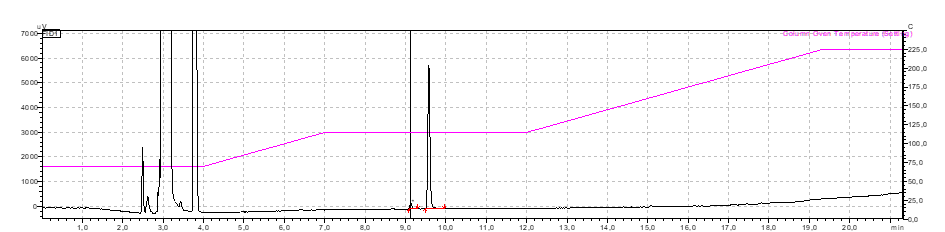


Figure S16: Chiral separation of enantiomers of 3-hydroxy-4-methylpentan-2-one 9a by GC. 9.1 min (*R*), 9.6 min (*S*), 92.5% *ee*.


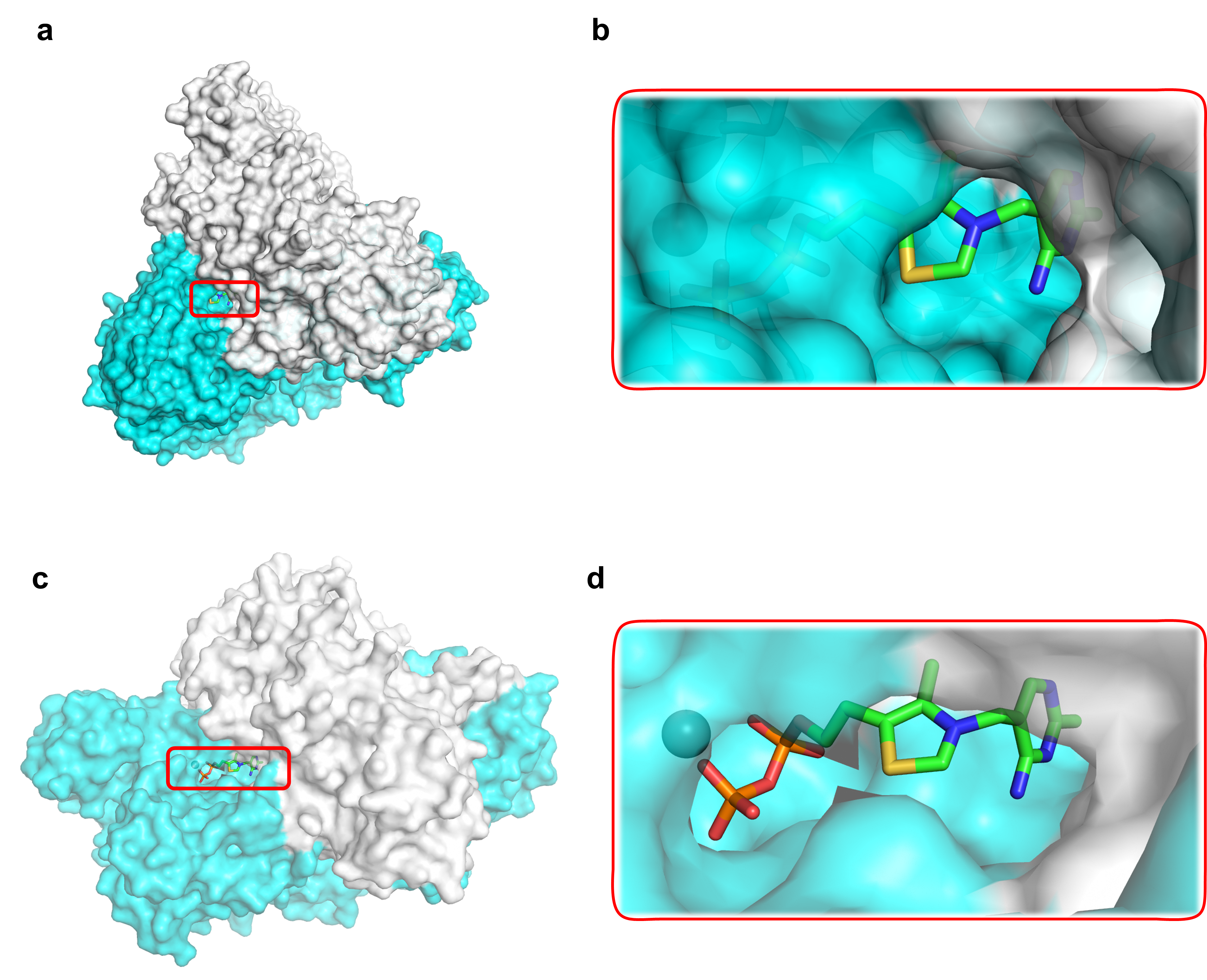


Figure S17: Structural comparison of WT *Sc*TK (1gpu.pdb) and WT EcPDH E1 (2iea.pdb) (a) Surface view of dimeric holo-*Sc*TK and (b) view of its narrow active site channel. (c) Surface view of dimeric holo-*Ec*PDH E1 and (d) view of its broad active site cleft. While both active sites are largely comprised of the same conserved residues (Figure 1, Figure 2, Figure 5, main manuscript), their overall structure differs considerably. This allows the active site of *Ec*PDH E1 to be overall more spacious.

**Michaelis-Menten curves**


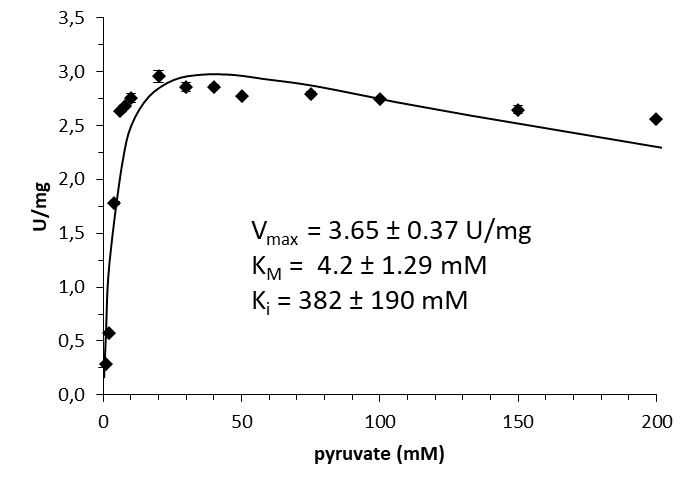


Figure S18: Kinetic analysis of sodium pyruvate, containing 0.1 mg/mL *Ec*PDH E1, 0.2 mM ThDP, 2 mM MgCl_2_, 50 mM glycolaldehyde, 20 mM KPi, pH 7.5, 37°C, *n =* 2.


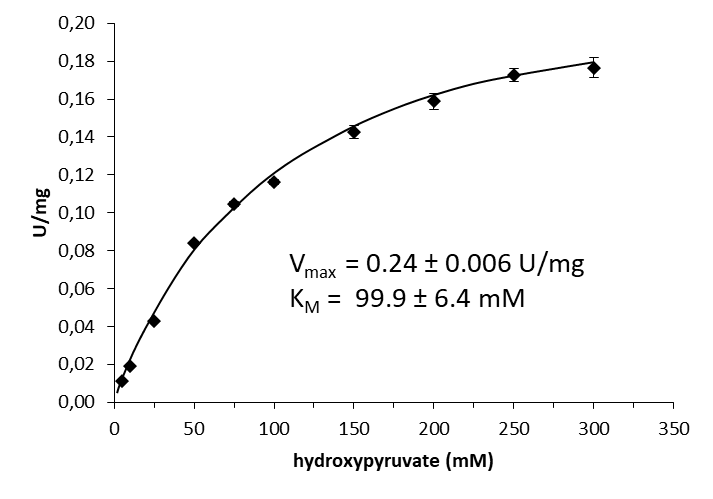


Figure S19: Kinetic analysis of lithium hydroxypyruvate, containing 0.1 mg/mL *Ec*PDH E1, 0.2 mM ThDP, 2 mM MgCl_2_, 50 mM glycolaldehyde, 20 mM KPi, pH 7.5, 37°C, *n =* 2.


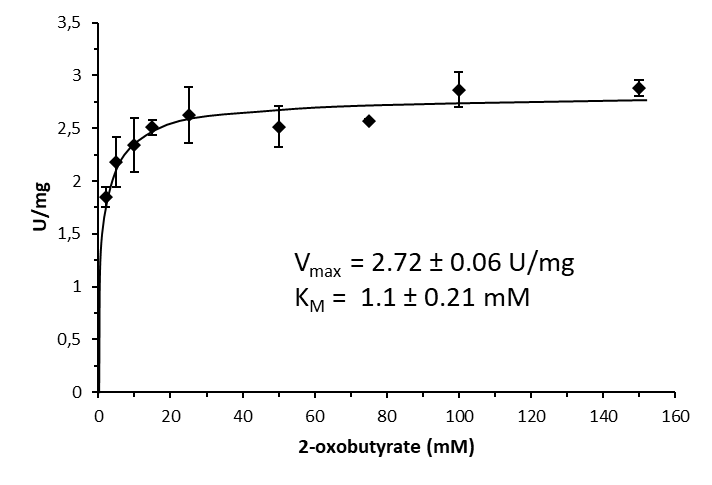


Figure S20: Kinetic analysis of sodium 2-oxobutyrate, containing 0.1 mg/mL *Ec*PDH E1, 0.2 mM ThDP, 2 mM MgCl_2_, 50 mM glycolaldehyde, 20 mM KPi, pH 7.5, 37°C, *n =* 2.


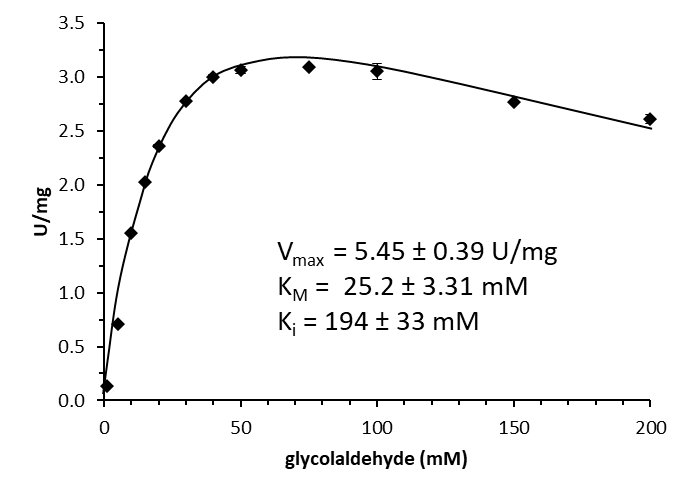


Figure S21: Kinetic analysis of glycolaldehyde, containing 0.1 mg/mL *Ec*PDH E1, 0.2 mM ThDP, 2 mM MgCl_2_, 50 mM sodium pyruvate, 20 mM KPi, pH 7.5, 37°C, *n =* 2.


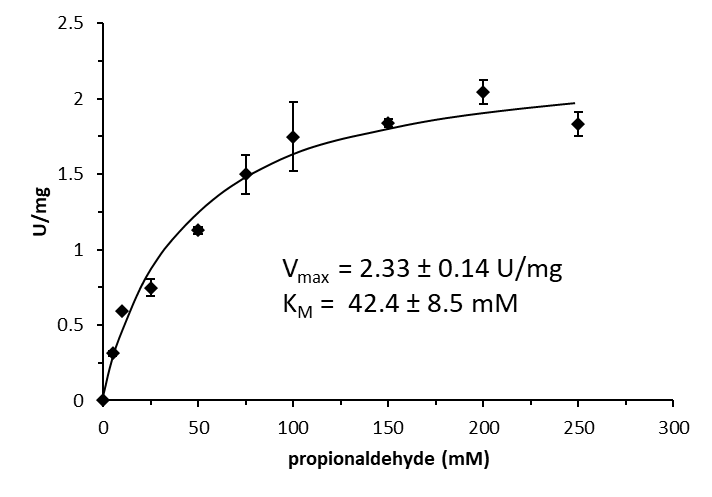


Figure S22: Kinetic analysis of propionaldehyde, containing 0.1 mg/mL *Ec*PDH E1, 0.2 mM ThDP, 2 mM MgCl_2_, 50 mM 2-oxobutyrate, 20 mM KPi, pH 7.5, 37°C, *n =* 2.


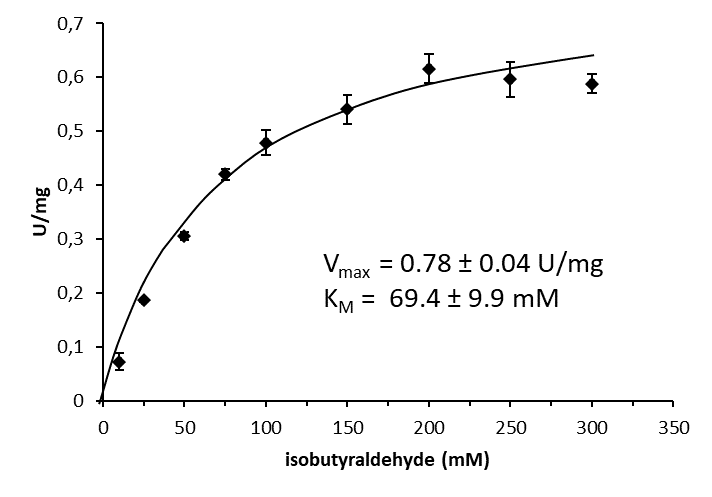


Figure S23: Kinetic analysis of isobutyraldehyde, containing 0.1 mg/mL *Ec*PDH E1, 0.2 mM ThDP, 2 mM MgCl_2_, 50 mM sodium pyruvate, 20 mM KPi, pH 7.5, 37°C, *n =* 2.

**NMR spectrums**


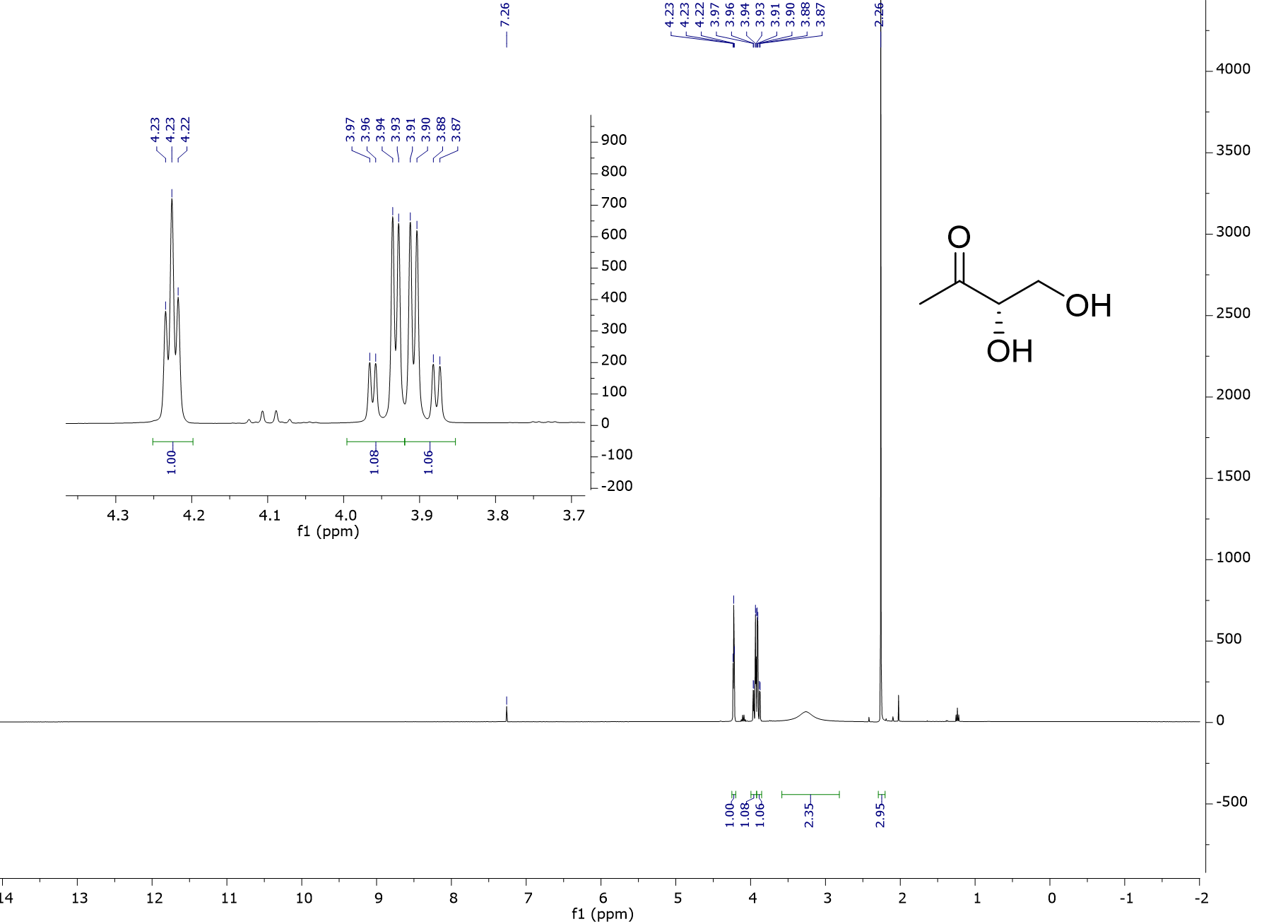


Figure S24: ^1^H-NMR of 4-deoxy-L-erythrulose 3a in CDCl_3_.


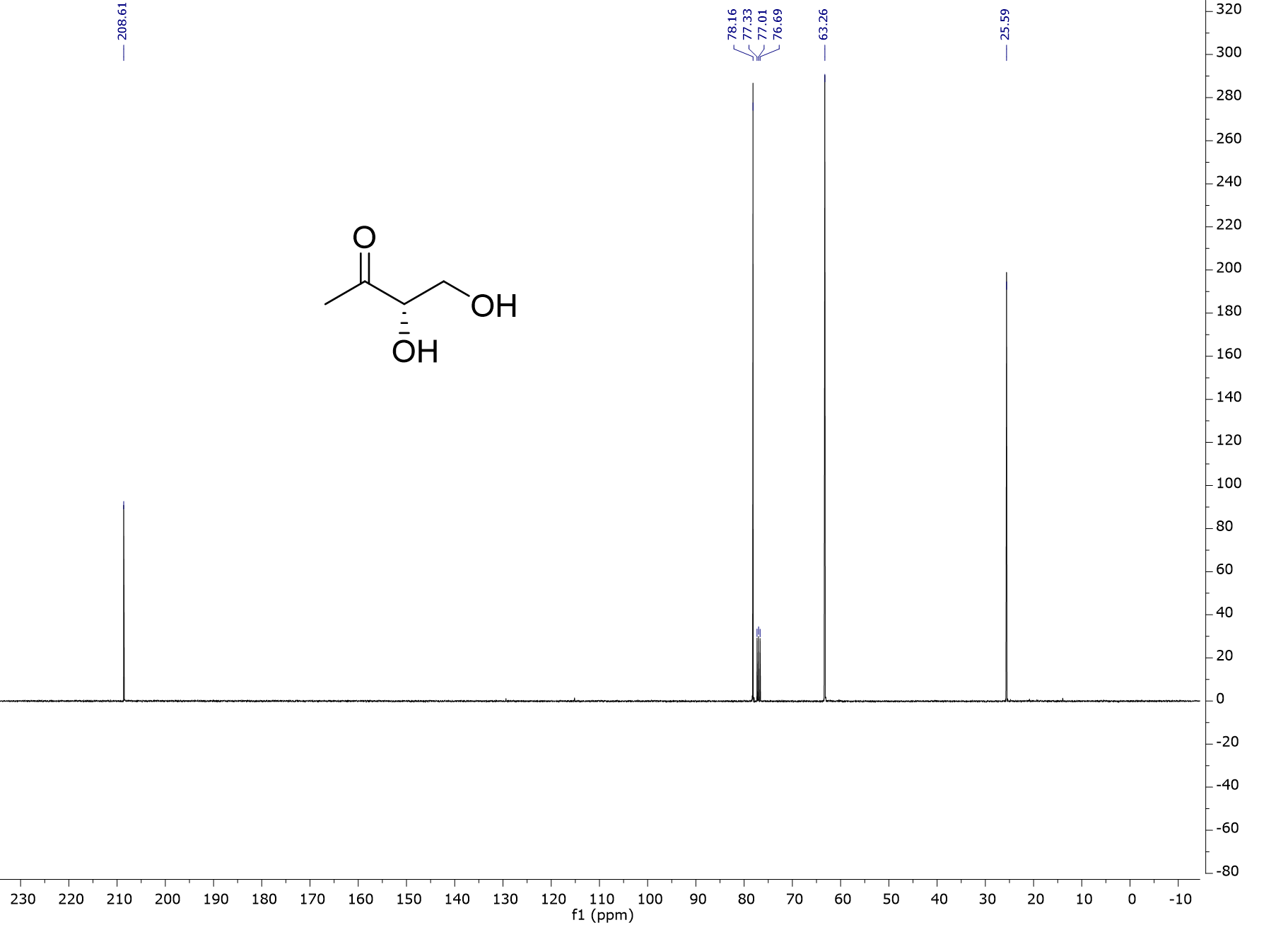


Figure S25: ^13^C-NMR of 4-deoxy-L-erythrulose 3a in CDCl_3_.


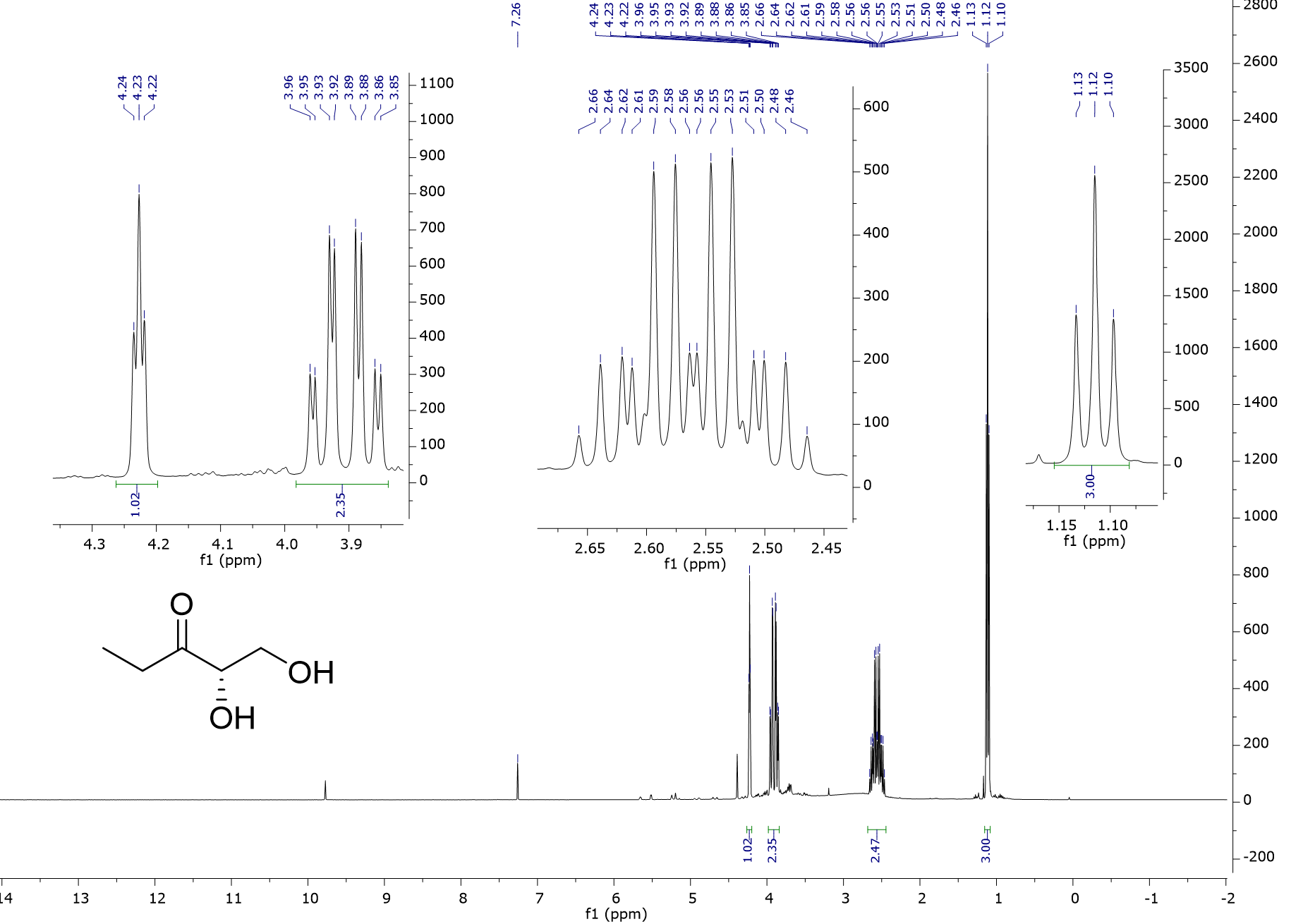


Figure S26: ^1^H-NMR of (*S*)-1,2-dihydroxypentan-3-one 3b in CDCl_3_.


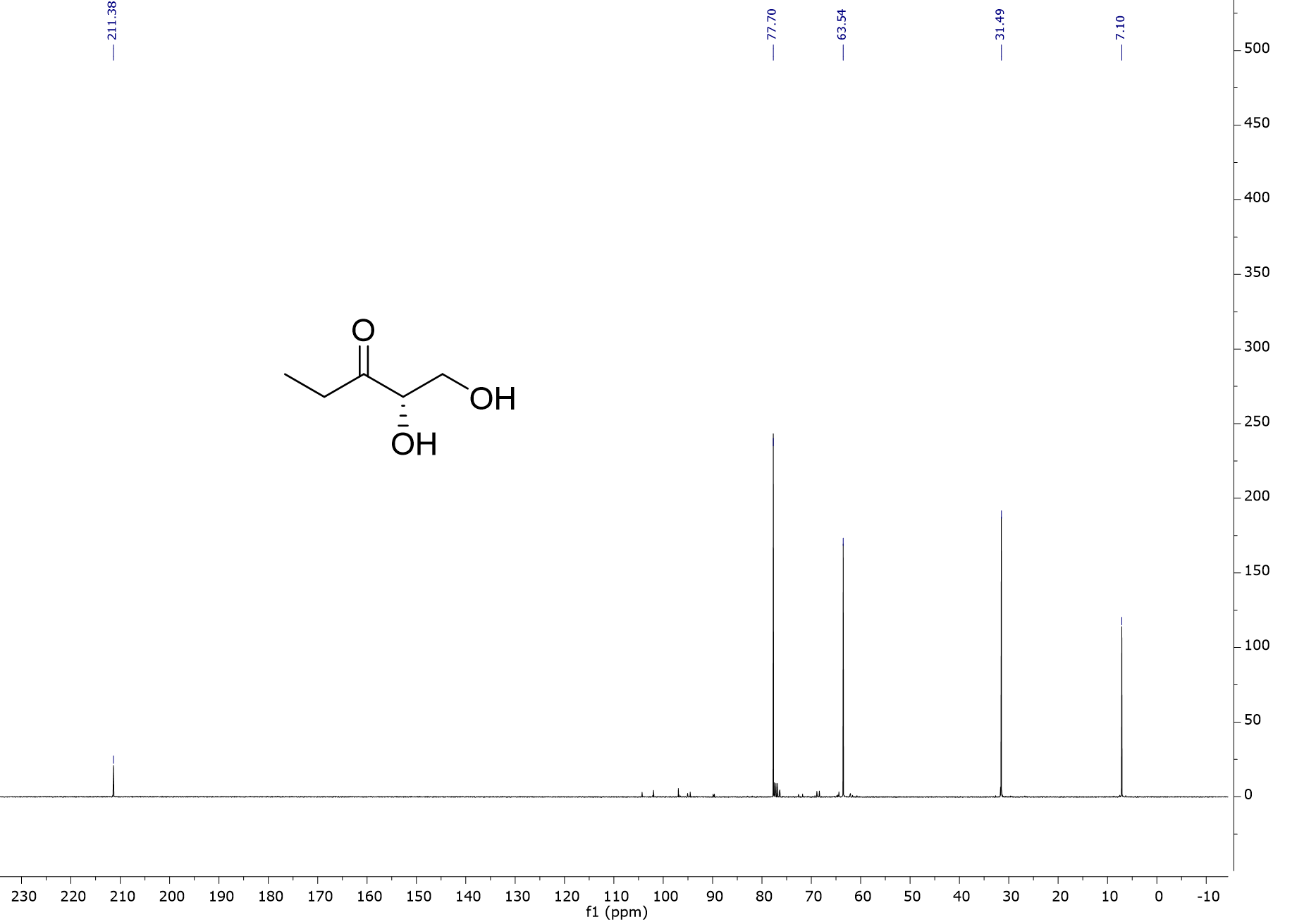


Figure S27: ^13^C-NMR of (*S*)-1,2-dihydroxypentan-3-one 3b in CDCl_3_.


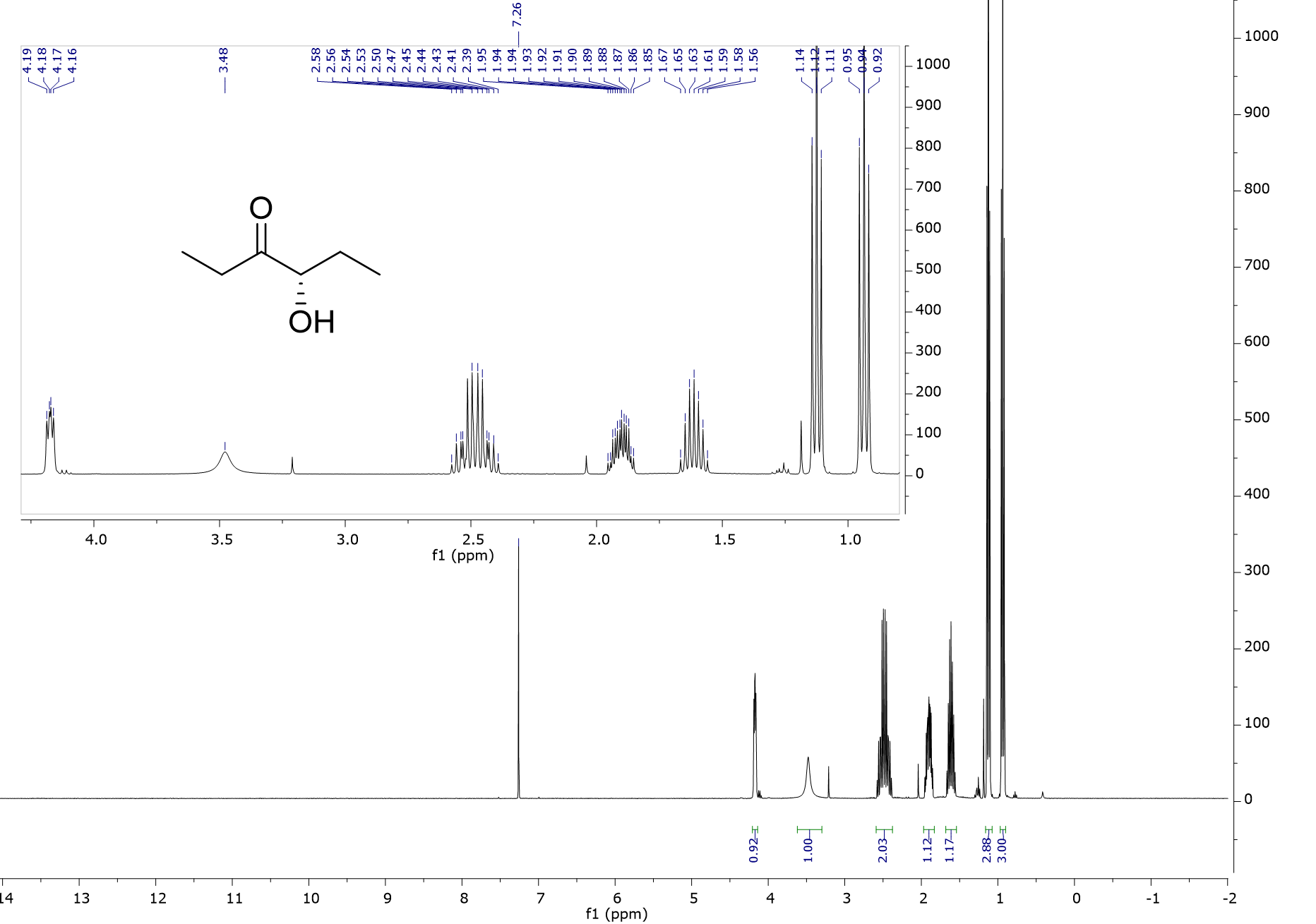


Figure S28: ^1^H-NMR of (*4S*)-hydroxyhexan-3-one 5b in CDCl_3_.


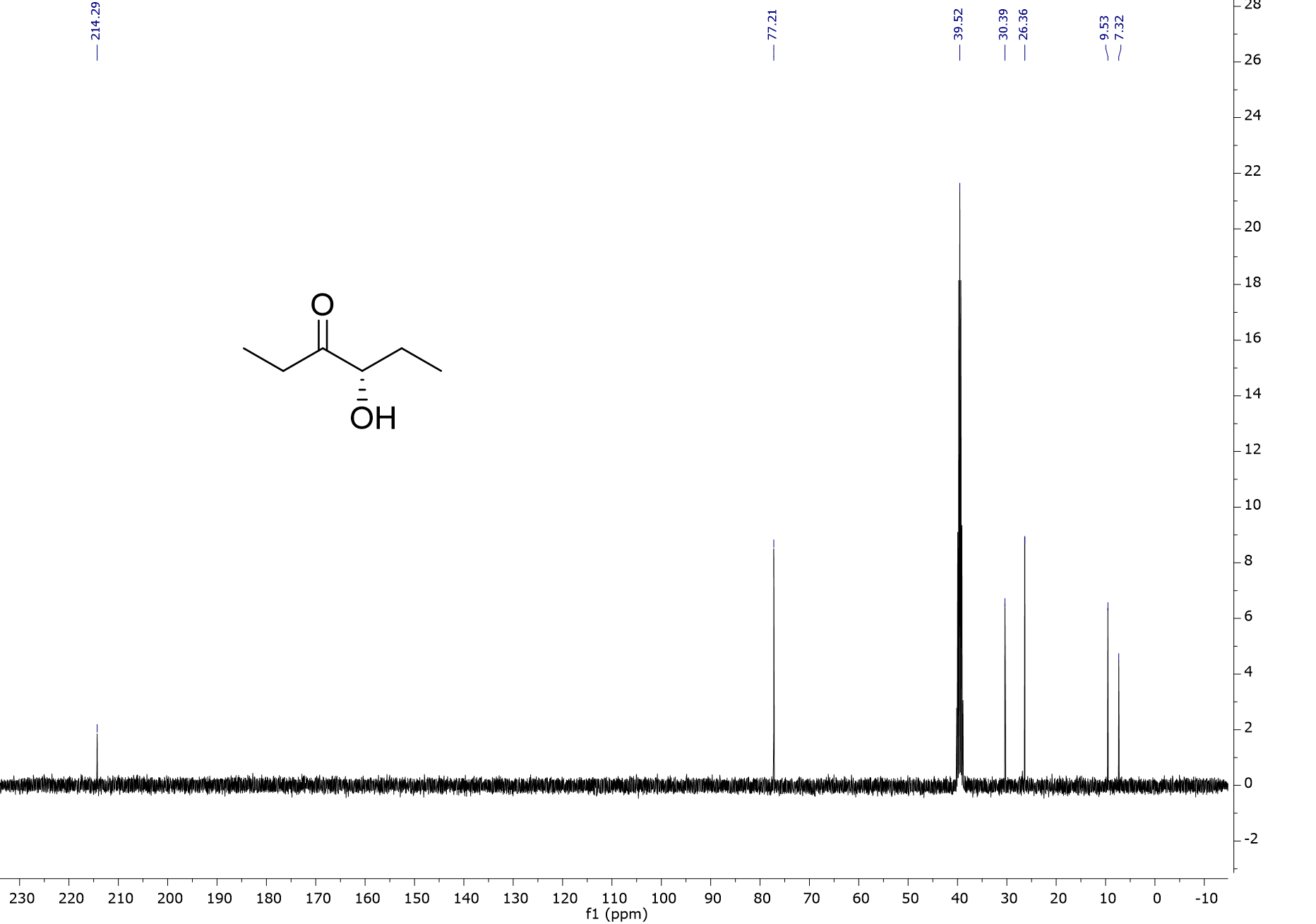


Figure S29: ^13^C-NMR of (*4S*)-hydroxyhexan-3-one 5b in DMSO-D_6_.


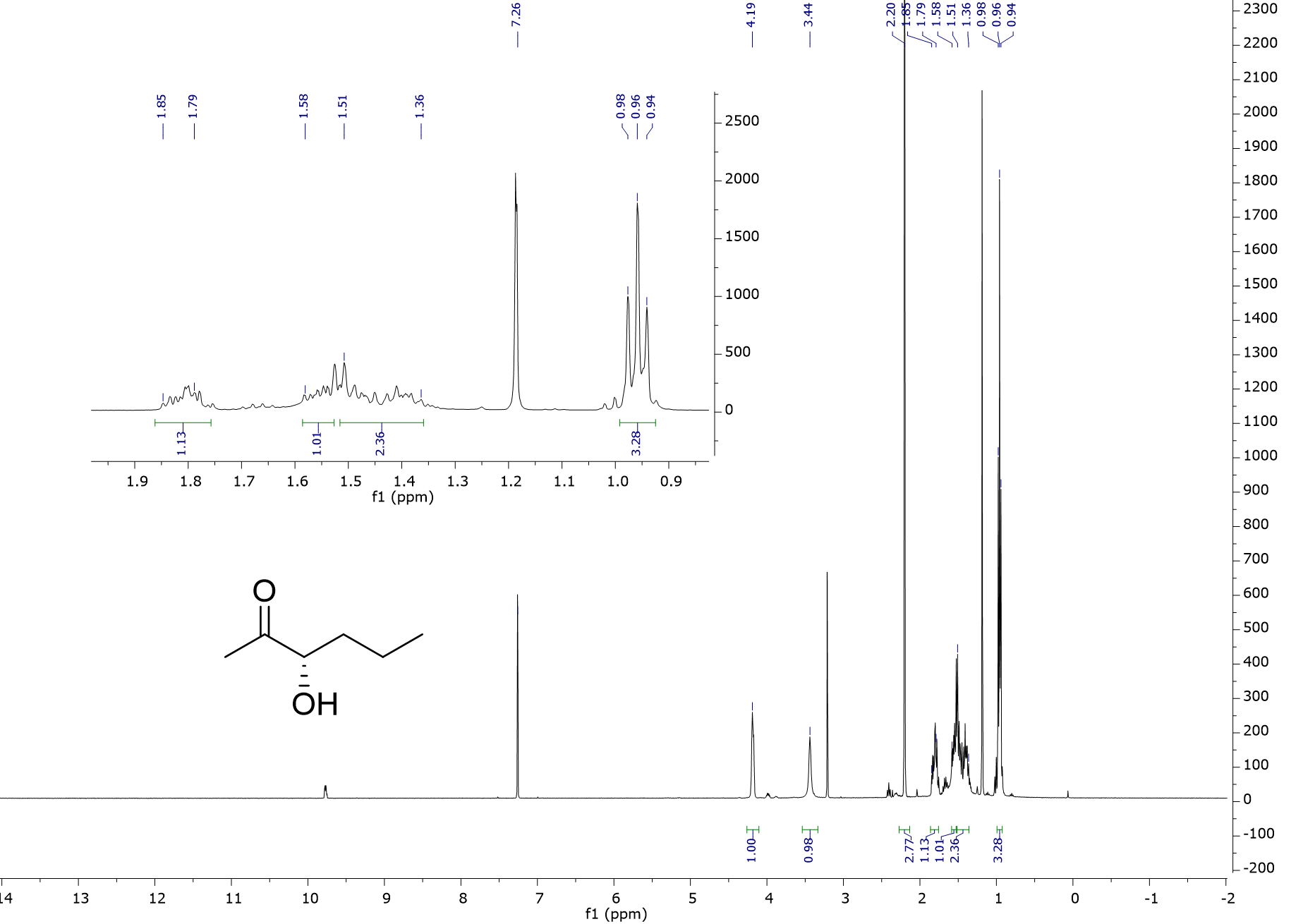


Figure S30: ^1^H-NMR of (*3S*)-hydroxyhexan-2-one 7a in CDCl_3_.


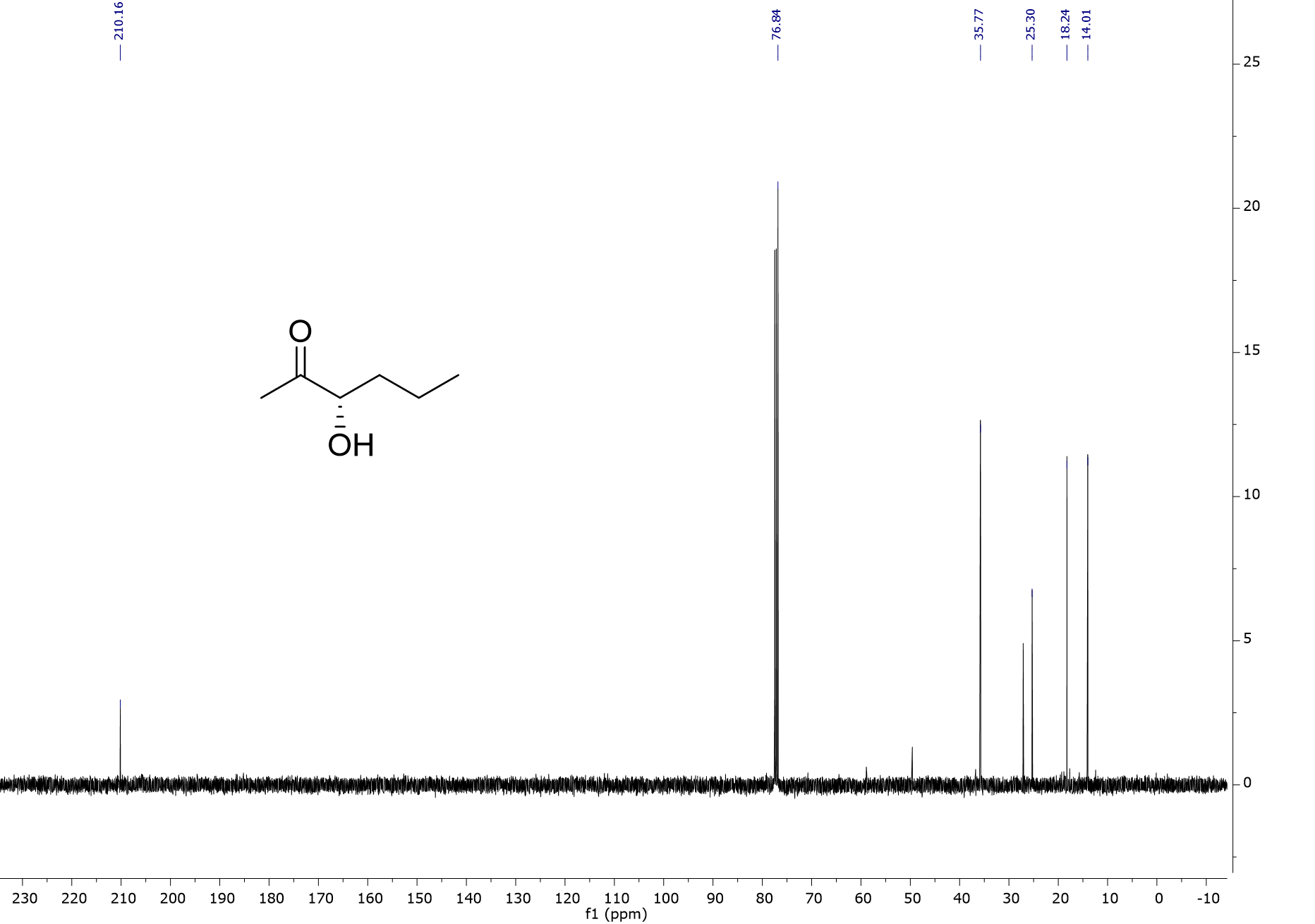


Figure S31: ^13^C-NMR of (3*S*)-hydroxyhexan-2-one 7a in CDCl_3_.


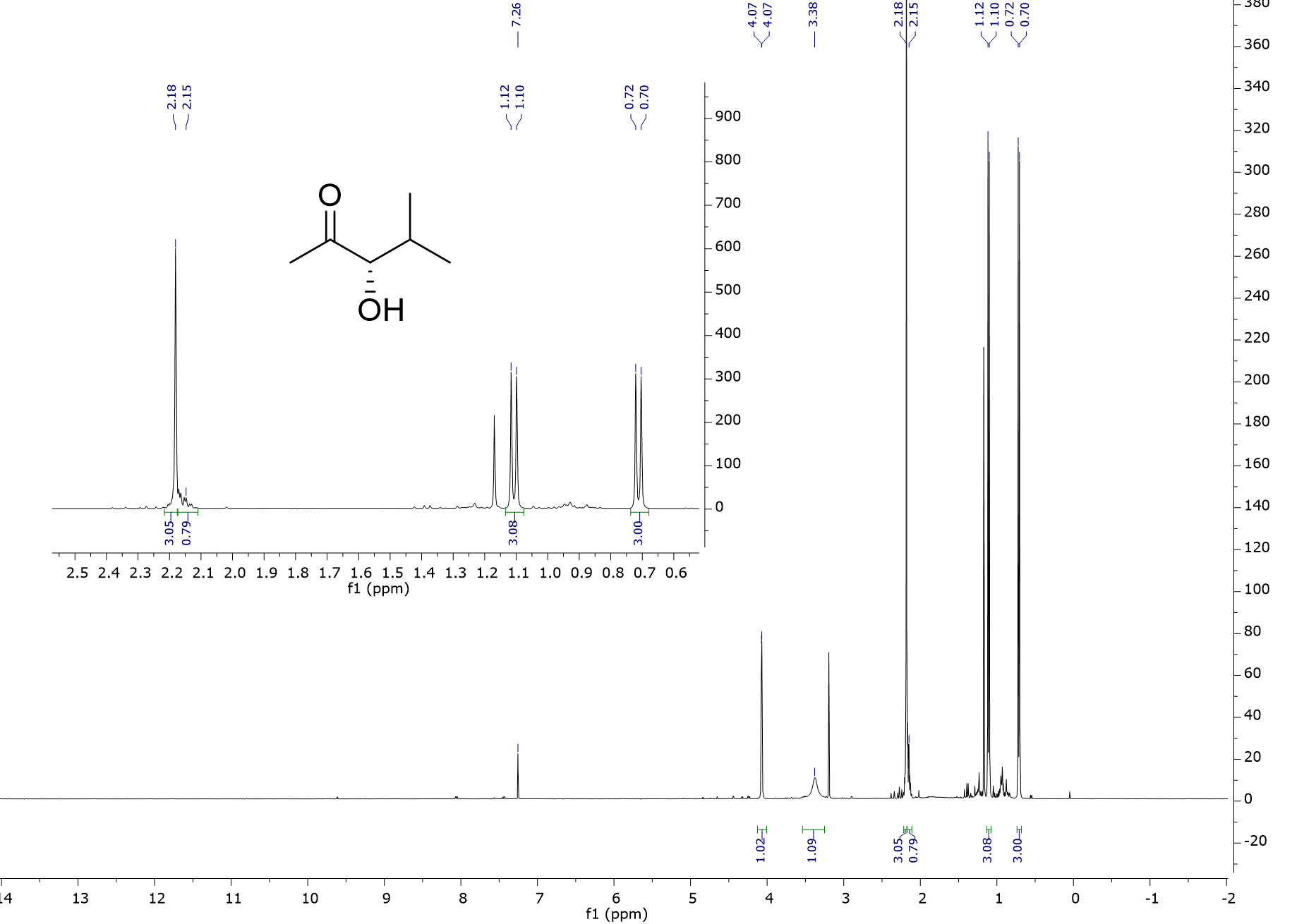


Figure S32: ^1^H-NMR of (*3S*)-hydroxy-4-methylpentan-2-one 9a in CDCl_3_.


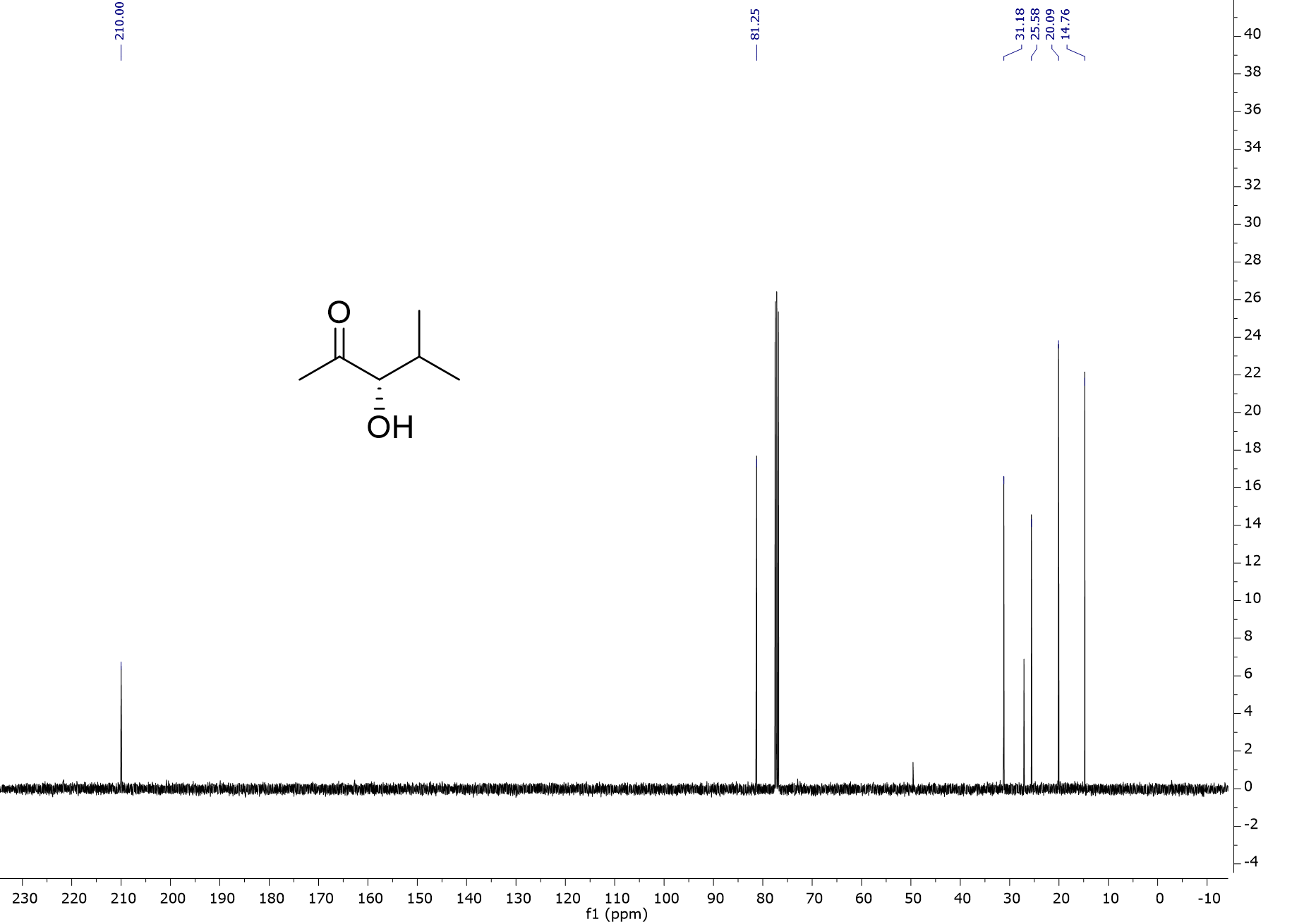


Figure S33: ^13^C-NMR of (3*S*)-hydroxy-4-methylpentan-2-one 9a in CDCl_3_.

Figure S34: Spectrophotometric calibration line for DCPIP, isosbestic point (517 nm, ε = 3.74 mM^-1^cm^-1^, *n =* 3).

Figure S35: Calibration line of 1-deoxy-erythrulose 3a (RP-HPLC, 210 nm, retention time = 10.2 min).

Figure S36: Calibration line of (*S*)-1,2-dihydroxypentan-3-one 3b (RP-HPLC, 210 nm, retention time =13.8 min).

Figure S37: Calibration line of L-erythrulose (RP-HPLC, 210 nm, retention time = 7.8 min).

Figure S38: Calibration of (*4S*)-hydroxyhexan-3-one 5b (RP-HPLC, 210 nm, retention time = 22.8 min).

**Figure S39:** Calibration line of (*3S*)-hydroxy-4-methylpentan-2-one **9a** (RP-HPLC, 210 nm, rt = 19.4 min).

Figure S40: Calibration line of sodium pyruvate (RP-HPLC, 210 nm, retention time = 7.8 min).

Figure S41: Calibration line of sodium 2-oxobutyrate (RP-HPLC, 210 nm, retention time = 10.6 min).

Figure S42: Calibration line of DL-glyceraldehyde (RP-HPLC, 210 nm, retention time = 8.4 min).
